# siRNA Knockdown of Ribosomal Protein Gene *RPL19* Abrogates the Aggressive Phenotype of Human Prostate Cancer

**DOI:** 10.1371/journal.pone.0022672

**Published:** 2011-07-22

**Authors:** Alix Bee, Daniel Brewer, Carol Beesley, Andrew Dodson, Shiva Forootan, Timothy Dickinson, Patricia Gerard, Brian Lane, Sheng Yao, Colin S. Cooper, Mustafa B. A. Djamgoz, Christine M. Gosden, Youqiang Ke, Christopher S. Foster

**Affiliations:** 1 Section of Cellular Pathology and Molecular Genetics, Department of Molecular and Clinical Cancer Medicine, Institute of Translational Medicine, University of Liverpool, Liverpool, United Kingdom; 2 Molecular Carcinogenesis Group, Institute of Cancer Research, Sutton, Surrey, United Kingdom; 3 Liverpool Microarray Facility, Centre for Genomic Research, University of Liverpool, Liverpool, United Kingdom; 4 Division of Cell and Molecular Biology, Imperial College London, London, United Kingdom; University of Louisville, United States of America

## Abstract

We provide novel functional data that posttranscriptional silencing of gene *RPL19* using RNAi not only abrogates the malignant phenotype of PC-3M prostate cancer cells but is selective with respect to transcription and translation of other genes. Reducing *RPL19* transcription modulates a subset of genes, evidenced by gene expression array analysis and Western blotting, but does not compromise cell proliferation or apoptosis *in-vitro*. However, growth of xenografted tumors containing the knocked-down *RPL19 in-vivo* is significantly reduced. Analysis of the modulated genes reveals induction of the non-malignant phenotype principally to involve perturbation of networks of transcription factors and cellular adhesion genes. The data provide evidence that extra-ribosomal regulatory functions of *RPL19*, beyond protein synthesis, are critical regulators of cellular phenotype. Targeting key members of affected networks identified by gene expression analysis raises the possibility of therapeutically stabilizing a benign phenotype generated by modulating the expression of an individual gene and thereafter constraining a malignant phenotype while leaving non-malignant tissues unaffected.

## Introduction

Ribosomal proteins (RPs) comprise a complex super-family of proteins [1] highly conserved throughout evolution, indicating their functional importance to living organisms [2]. This assertion is supported by the number of RP pseudogenes and gene duplications together with shared regions of identity between homologous proteins in prokaryotes and eukaryotes [3]. Eukaryotic ribosomes contain approximately 80 RPs together with four ribosomal RNAs (rRNA) and require some 150 non-ribosomal factors to become organized into their constituent small (40S) and large (60S) subunits [4]. Initially considered to be involved only in protein synthesis, certain RPs are recognized as pleiotropic and to mediate a variety of extra-ribosomal regulatory functions [5], [6]. Such RPs, include L5 [7], L11 [8], L13 [9] and S7 [10]. In zebrafish (*Danio rerio*) a powerful role for RPs as tumor-suppressors has been demonstrated whereby mutation or suppression in any of several RP genes impairs control of p53, thus promoting malignancy [11], [12]. Recently, the concept of “ribosomopathy” has become established whereby mutation of a particular RP is pathogenic for a specific disease [13]. Approximately 25% of cases of Diamond-Blackfan anemia are caused by mutation of ribosomal protein gene *RPS19* while in another 20%, mutations occur in other ribosomal protein genes [14]. Currently, some 77 individual *RPS19* mutations have been described [15]. In addition, haploinsufficiency for ribosomal proteins has been shown, in some cases, to be an underlying cause for Diamond Blackfan anemia [16].

Presently, mechanisms relating mutations in RP genes to cancer remain unknown [17]. For the proximal long arm region of chromosome 17 where the *RPL19* gene is located (17q), major cancer-specific changes have been described. These include amplifications and copy number changes, particularly those of the region that include oncogene *ERBB2*, formation of isochromosome 17q, duplications, deletions, mutations and other genomic rearrangements. Previously [18], we identified enhanced expression of *RPL19* mRNA in prostate cell-lines and tissues to correlate with an aggressive malignant phenotype. Since elevated *RPL19* mRNA occurred as one of a relatively small number of sequences over-expressed in prostate cancer, we hypothesized that its effect was likely to be selective rather than part of a global non-specific elevation in gene expression. Ribosomal protein L19e (RPL19) belongs to the L19E super-family of proteins and, in eukaryotes, is a component of the ribosomal large 60S subunit. The gene is expressed throughout much of evolution, particularly in eukaryotes and archaea but is absent from bacteria [19], [20] although there is homology between sequences in rat L19 and *E.coli* ribosomal proteins L18, L30 and S2 [21] Surprisingly, for such an apparently important gene, *RPL19* has thus far received little attention. In humans, *RPL19* maps on chromosome 17 at 17q11.2–q12 where it encodes 9 potential splice variants. In a series of human breast cancer biopsies, *RPL19* has been reported as being expressed and co-amplified together with *ERBB2* and genes *PNMT*, *PSMB3* and *NR1D1* [22]. This complex region containing multiple genes has been suggested as a possible amplicon [23], [24] extending for some ∼547 kb from *RPL19* through *STRAD3* and *ERBB2* to *GRB7* in the region 17q11.2–q12. Presently, no data have substantiated this speculation. In prostate cancer, amplification of erbB2 is infrequent, being reported in only 0.04% [25] to 2% [26] of cases, and therefore not a common mechanism of RPL19 over-expression. Since our initial identification of RPL19 in prostate cancer [18], its expression has been shown to define poor-prognosis colorectal cancer [27] and as a novel tumor antigen in lung adenocarcinoma [28].

Global changes in genes modulated in human prostate cancer have previously been profiled using DNA expression array analysis [29] that have detected changes in gene expression following selective up-regulation of individual target genes [30], [31] or following gene-knockdown using antisense [32] or RNAi [33] technology with subsequent transformation of the malignant phenotype. The differentially-expressed genes and their associated networks have been assessed as biomarkers to segregate different prostate cancer phenotypes according to behavior and response to therapy [34]. However, an altered level of gene expression does not, *ipso facto*, confirm a primary role in the malignant process. Genomic instability is the hallmark of malignant transformation [35] and the effects of gain or loss of a single gene are likely to be transmitted throughout the genome with the consequence that expression of other genes becomes secondarily modulated [36]. Such changes either may have immediate and active relevance to the resulting cellular phenotype or their altered expression is passive and inconsequential. To assess the functional relevance of a particular gene, suppression of its transcription allows analysis of its immediate effects on genome-wide expression. Previously, we have transfected malignant prostatic epithelial PC-3M cells with a 436 bp-long antisense oligonucleotide to knock-down expression of *FABP5* that ameliorated the malignant tumor phenotype both *in-vitro* and *in-vivo* [37]. herein, we have employed the more surgical technique of RNA interference (RNAi) with potentially greater specificity and efficiency, depending upon the particular gene being targeted [38].

Our previous data [18] indicated that expression of *RPL19* might be functionally important in promoting prostatic malignancy. We have now tested this hypothesis by selectively reducing *RPL19* expression using RNAi. The resulting PC-3M cells exhibit an abrogated malignant phenotype both *in-vitro* and *in-vivo* when submitted to phenotypic assessment and gene expression analysis. The data support the possibility of a functional role for *RPL19*, acting within a spectrum of altered gene expression, in maintaining the malignant phenotype of human prostate cancer cells. Confirmation of such a scenario would allow selective therapeutic targeting of RPL19, either immunologically [28] or using small molecules, to modulate discrete subsets of cellular proteins that are key promoters of the malignant phenotype.

## Results

### siRNA knockdown of RPL19 in parental PC-3M cells

#### Transient transfection

qPCR analysis of the parental PC-3M cells using the primers defined in Table 1 revealed strong *RPL19* mRNA expression, confirmed by nucleotide sequencing.

**Table 1:** Primer sequences employed for qPCR identification and quantification of mRNAs.

Primer	Direction	Sequence	Amplicon Size
*RPL19*	Forward	GGGCATAGGTAAGCGGAAGG	149
	Reverse	TCAGGTACAGGCTGTGATACA	
Human β-actin	Forward	AGCCTCGCCTTTGCCGA	174
	Reverse	CTGGTGCCTGGGGCG	
stromelysin 1 (*MMP3*)	Forward	AAATCCCTCAGGAAGCTTGA	137
	Reverse	GCCCAGAATTGATTTCCTTT	
stromelysin 2(*MMP10*)	Forward	CAGGACACAGTTTGGCTCAT	101
	Reverse	GGTGCCTGATGCATCTTCT	
collagenase 3(*MMP13*)	Forward	GGCAAACTTGACGATAACAC	139
	Reverse	GGGTGTAATTCACAATTCTGTAGG	
Fas (TRNF6) associated factor 1 (*FAF1*)	Forward	CTTCAGCGTTTCGACCTGTA	225
	Reverse	GGACCGTACTGTCTTCCACA	
nuclear factor of kappa light polypeptide gene enhancer in B-cells inhibitor, alpha (*NFκBIA*)	Forward	TCCGAGACTTTCGAGGAAAT	143
	Reverse	ACACGTGTGGCCATTGTAGT	
stonin 2 (*STON2*)	Forward	AGCAACTGGGTTCAGTTTGA	90
	Reverse	GGTCAATGGTAGGGCTGTCT	

**Figure 1:**
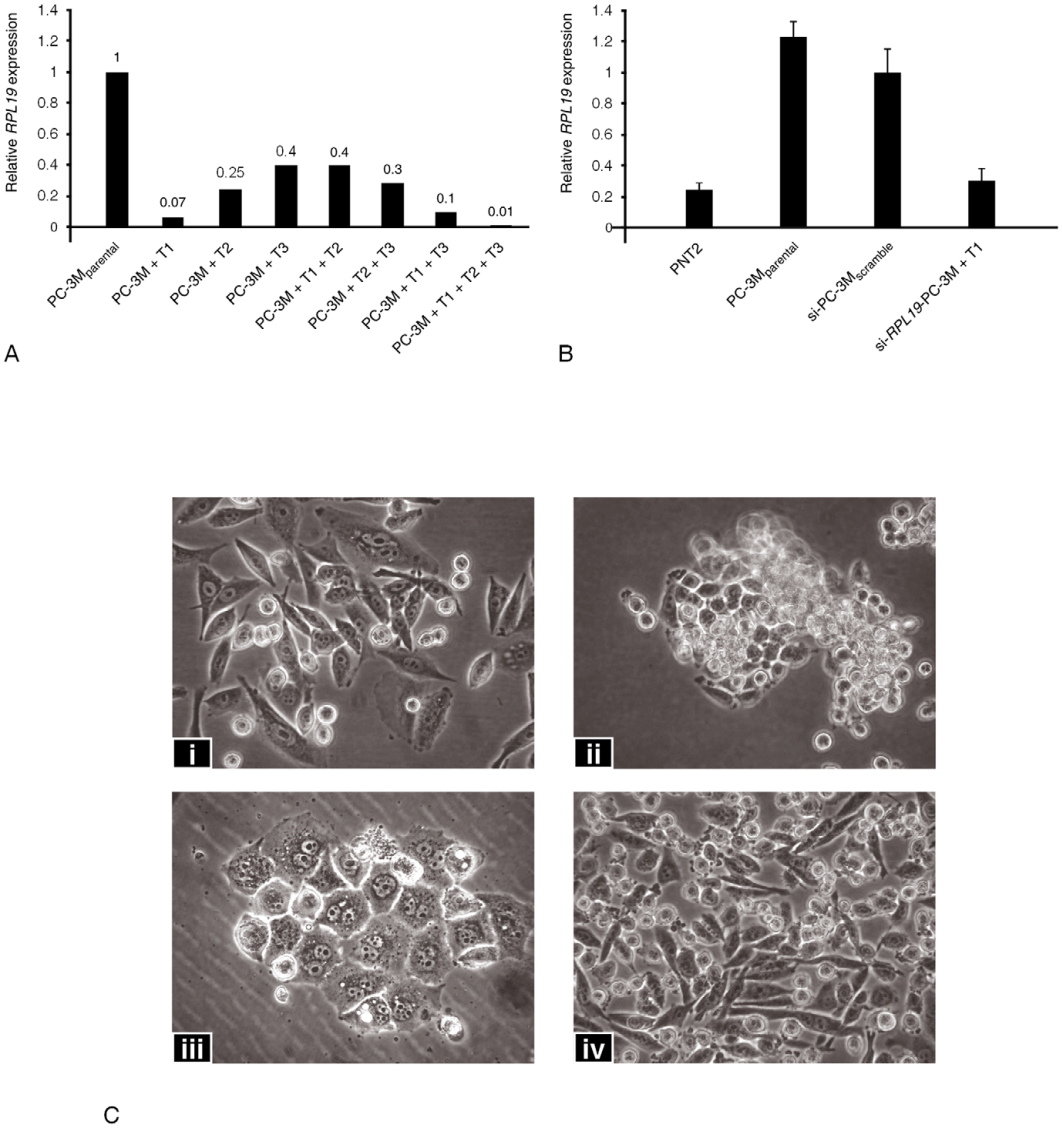
Effect of silencing *RPL19* relative to PC-3M_parental_ cells. A. qPCR analysis of *RPL19* expression levels following transient silencing of different targets in PC-3M_parental_ cells. Target #1 (T1) was the most efficient with only 7% residual level detected. This reduction was only exceeded by the simultaneous combination of T1+T2+T3. B. qPCR analysis of *RPL19* expression levels following stable silencing of Target #1. These data are compiled from experiments performed in triplicate. Measurements are relative to the expression of RPL19 in si-PC-3M_scramble_ cells. Comparative levels in benign PNT2 cells are also shown. C. Morphological appearances of (i) PC-3M_parental_ cells and various of the colonies (ii-iv) following stable knockdown of *RPL19*. Some colonies (ii) were poorly adherent with the majority of cells growing in suspension. Others (iii) contained predominantly multinucleate forms. The majority (iv) comprised cells that were smaller than the parental. Clone ST-3 cells used in all subsequent experiments are shown in this panel. (Magnification ×200)

Thereafter, transient transfection of siRNA sequences to *RPL19* exon 1 (Table 2) revealed Target #1 to be the most effective sequence for RNA silencing, reducing its expression to only 7% of its initial level (Figure 1A). While the other sequences were effective, only the combination of all three simultaneously was better than Target #1, alone. Thereafter, Target #1 was used for all subsequent experiments.

**Table 2:** Details of potential target sequences to silence *RPL19* in PC-3M cells.

Target	Sequence	Position in gene sequence	Exon silenced	Variants affected
#1	AAGCTCATCAAAGATGGGCTG	15	11	a, b, c, d, e, g
#2	AAACAAGCGGATTCTCATGGA	43	18	a, c, d, f, h
#3	AAGATACCGTGAATCTAAGAA	86	15	a, b, c, d, e

This Table identifies the particular exon silenced and the alternative splice variants predicted to be affected.

#### Stable transfection

Levels of *RPL19* mRNA were measured in PNT2, PC-3M_parental_, PC-3M_scramble_ and si-*RPL19-*PC-3M_target #1_ transient transfectant cells (Figure 1B). In accordance with the previous study [33], expression in PC-3M_scramble_ cells was set at unity and relative expressions in the other cell-lines were compared as fold-differences. *RPL19* expression in PC-3M was 4.9 times greater than that of the PNT2 cells and consistent with our previous studies confirmed by Northern blot analysis [18]. In the si-*RPL19-*PC-3M_clone ST-3_ transfectant cells, expression of *RPL19* was reduced to only 1.3 times greater than the PNT2 cells. PC-3M_scramble_ cells revealed a 2.3 fold reduction in *RPL19* when compared to PC-3M_parental_, although this value was not statistically significant. Single cell cloning [33] followed by qPCR and Western blotting confirmed si-*RPL19-*PC-3M_clone ST-3_ expressed the lowest levels of *RPL19* mRNA and protein. This clone of cells was thereafter employed for detailed phenotypic analysis.

### Growth characteristics of si-RPL19cells *in-vitro*

**Figure 2:**
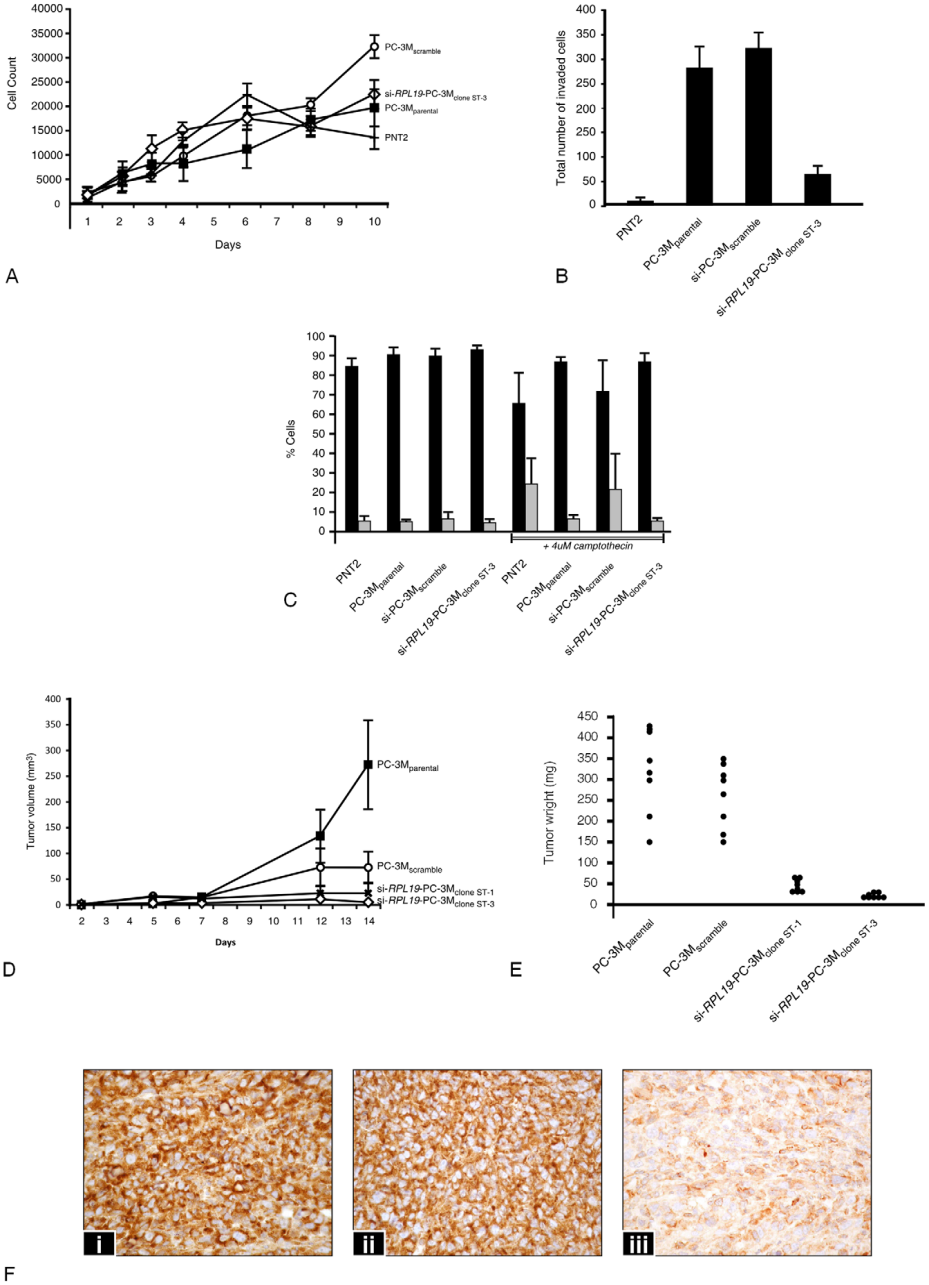
Growth characteristics of si-PC-3M_clone ST-3_ cells *in-vitro* and *in-vivo*. A. Relative growth of cell-lines in monolayer culture revealing no statistical difference in the rate of proliferation between the knockdown cells (si-*RPL19*-PC-3M_clone ST-3_) and that of PC-3M_parental_ cells. B. Invasion assay *in-vitro* comparing the same populations of cells as those shown in (A) and revealing an 83% decline in the invasive capacity of the *RPL19* knockdown cells relative to PC-3M_scramble_ cells. C. Resting levels of apoptotic indices were not significantly different in the benign (PNT2), parental (PC-3M) or knockdown cells. After challenge by camptothecin, no change was identified in the PC-3M_parental_ or si-*RPL19*-PC-3M_clone ST-3_ cells. While an increase in apoptosis was found in the benign cells and in the scramble-transfected cells, these were not significant. D. Growth of tumor cells *in-vivo* by estimated volume revealed a highly significant (*p*<0.005) suppression of growth by two of the stable transfectant clones, when compared to the PC-3M_parental_ and PC-3M_scramble_ cells. Growth of PNT2 cells is not included since we have already shown [33] the growth of tumors to be infrequent, particularly over the time-span of these experiments. E. Analysis of tumor weights *in-vivo* confirmed clones ST-1 and ST-3 to generate tumors significantly (*p*<0.005) smaller than the PC-3M_parental_ or the si-PC-3M_scramble_ cells. F. Immunohistochemical analysis of tumors growing as xenografts *in-vivo* supported the mRNA levels data (Figure 1B) that whereas the original PC-3M_parental_ (i) and si-PC-3M_scramble_ (ii) cells expressed RPL19 protein at high level. The si-*RPL19*-PC-3M_clone ST-3_ cells (iii) expressed RPL19 heterogeneously and at only very low levels. (Magnification ×350)

Clones of transfected si-*RPL19*-PC-3M cells grown under standard conditions exhibited differences in morphology (Figure 1C). Compared to PC-3M_parental_ cells, si-*RPL19*-PC-3M cells were generally less adherent to substrate. However, these cells maintained an ability to proliferate and could be successfully sub-cultured, although a large proportion of the cells remained in suspension. Other si-*RPL19*-PC-3M cells showed an increase in multinucleate forms, suggesting impaired completion of mitosis. Proliferation assays (Figure 2A) revealed that during the logarithmic phase of growth, the rate of cell division by the si-*RPL19-*PC-3M_clone ST-3_ transfectant cells was not significantly affected (*p*≥0.05) when compared to PC-3M_parental_ and si-PC-3M_scramble_. The ability of si-*RPL19-*PC-3M_clone ST-3_ cells to invade an extracellular collagenous matrix (ECM) was compared to that of the PNT2, PC-3M_parental_ and PC-3M_scramble_ cell-lines (Figure 2B). The number of cells that invaded through the ECM were: (PNT2) 0.6±0.6, (PC-3M_parental_) 279 ± 33.7 and (PC-3M_scramble_) 317 ± 28.3 (*p*<0.001). The si-*RPL19*-PC-3M cells exhibited a comparatively poor invasive potential at only 60 ± 10.7 transmigrating cells (*p*<0.001). Thus, silencing *RPL19* reduced the invasive potential of PC-3M cells approximately 5-fold. Endogenous (basal) levels of apoptosis within the PC-3M_parental_ and PC-3M_scramble_ cells (Table 3 and Figure 2C) were similar to those obtained during comparable studies of the *PRKCZ* gene [33]. Basal levels of apoptosis in the four cell-lines were not statistically different (*p*>0.05). Although sensitivities of the PC-3M_parental_ and si-*RPL19-*PC-3M_clone ST-3_ cells to camptothecin were not altered, this agent increased apoptosis in PNT2 and PC-3M_scramble_ cells (*p*<0.0001).

**Table 3:** Comparative effects of *RPL19* knockdown on apoptotic rates in prostate cells.

Cell-line	Basal level of apoptosis (%)	Level of apoptosis following camptothecin (%)	Student's t test (*p*)
PNT2	5.68±3.4	24.5±1.4	<0.0001
PC-3M_parental_	5.53±1.4	4.74±1.4	>0.05
PC-3M_scramble_	7.6±1.9	21.5±1.4	<0.001
si-*RPL19*-PC-3M_clone ST-3_	3.9±0.9	4.43±1.4	>0.05

### Tumorigenicity and RPL19 protein expression *in-vivo*

In all groups of animals, tumors became apparent on day 2 following inoculation (Table 4). However, more appeared sooner in the PC-3M_parental_ (3/8) and PC-3M_scramble_ (4/8) groups. In the two transfectant clone groups, tumors took longer to appear (2/8 tumors in animals carrying the si-*RPL19-*PC-3M_clone ST-3_ cells and 1/8 tumors in animals carrying the si-*RPL19-*PC-3M_clone #2_ cells). Initially, all tumors were similar in size. After 7 days the PC-3M_parental_ and PC-3M_scramble_ groups developed larger tumors than two transfectant groups (Figure 2D). At autopsy, 15 days after inoculation, a significant difference (*p*<0.001) was apparent in the mean weights of the control and *RPL19-*knockdown tumors (Figure 2E). PC-3M_parental_ exhibited a wide range in tumor weight, one animal producing a tumor of 810 mg in 15 days, the maximum allowed by the Project License. Conversely, another animal developed a tumor of only 10 mg. A similar phenomenon occurred within the PC-3M_scramble_ group with tumors ranging from 10-140 mg. The final weights of the PC-3M_parental_ tumors were not significantly different from those of the PC-3M_scramble_ group (Mann-Whitney U Test, *p*>0.05). Thus, si-RNA suppression of *RPL19* affected the size of the tumors generated *in-vivo* (*p*<0.05) but not on their latency. No micrometastases were identified at autopsy or on subsequent histopathological examination of the excised tissues.

**Table 4:** Incidence and latency period of tumors produced by transfectants in nude mice.

Cell-line	No of animals inoculated	Incidence of tumors*		Median latent period in days (range)	Mean weight of tumors (mg.)**
		No	%		
PC-3M_parental_	8	8	100	5.25 (2–12)	337.5±266.5
PC-3M_scramble_	8	8	100	4.275 (2–12)	120±83.7
si-*RPL19*-PC-3M_clone ST-1_	8	8	100	6.875 (2–12)	36.0±35.0
si-*RPL19*-PC-3M_clone ST-3_	8	4	50	4.25 (2–5)	7.5±13.9

* Tumor incidence is the percentage of mice with tumors/total number of inoculated animals.

** The final weights of the si-*RPL19*-PC-3M_clone_ tumors were significantly less than the PC-3M_parental_ and the PC- 3M_scramble_ tumors (Mann-Whitney U Test, p<0.05).

Immunohistochemistry of tumor xenografts detected strong expression of RPL19 protein in both the PC-3M_parental_ and si-PC-3M_scramble_ cells (Figure 2F). Knockdown cell lines si-*RPL19-*PC-3M_clone ST-3_ and si-*RPL19-*PC-3M_clone ST-1_ exhibited comparatively little staining, indicating continued suppression of the *RPL19* gene in the majority of tumor cells. Detection of small amounts of RPL19 protein in some tumor cells is considered to represent clonal variation resulting from continued low-level expression of the gene, rather than its total inhibition, as identified by qPCR of the cells *in-vitro* and the results of the Western blotting studies. While expression of mRNA and corresponding protein in prostatic epithelium are not always concordant [39], apparent discrepancies between *in-vitro* and *in-vivo* studies may be due to the *in-vivo* effects of a surrounding stromal matrix affecting tumor cell adhesion or to other influences including growth factors modulating individual low-level gene expression [40]–[42].

### Comparative gene expression profiling of si-RPL19-PC-3M_clone ST-3_ cells

**Figure 3:**
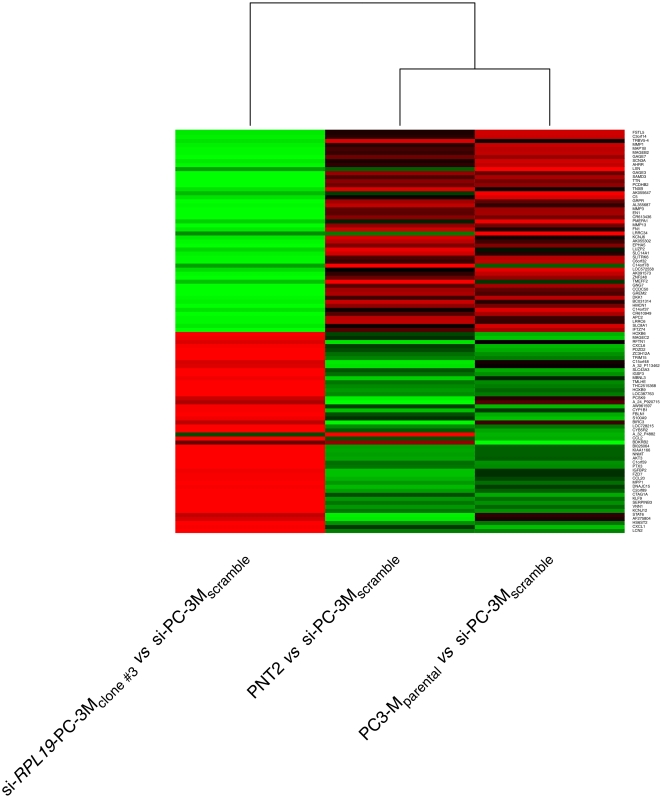
Graphical representation of gene expression modulated following *RPL19* knockdown. Heat map of top 50 genes up-regulated and top50 genes down-regulated following expression-profiling of mRNA expressed by si-*RPL19*-PC-3M_clone ST-3_ cells when compared to PC-3M_parental_ cells using PC-3M_scramble_ cells as the common denominator. Hierarchical clustering is shown. Green indicates genes over-expressed in a sample compared to scramble-transfected cells. Red indicates genes down-regulated in the sample when compared to scramble-transfected cells. Corresponding numerical data are presented in Supporting Information Tables S1 & S2.

Genome-wide expression profiles obtained from DNA oligonucleotide microarrays (unmodified Agilent Human Genome 44K) were employed to identify genes modulated following *RPL19* knockdown. Comparison of genes expressed by PC-3M_parental_ and PC-3M_scramble_ cell-lines revealed no statistically significant differences (*p*≥0.05), indicating that the transfection technique was not responsible for appreciable off-target effects that might bias the experimental data. A total of 916 DNA sequences, representing 768 genes, were identified as differentially expressed (*p*≤0.05, Benjamini and Hochberg multiple testing correction applied). Of these, 404 were enhanced and 364 down-regulated. Within that data set, 184 different genes were modulated at least four-fold, 62 being up-regulated and 122 down-regulated. The top 50 differentially-expressed genes in these two categories are summarized in Supporting Information Tables S1 and S2 and graphically (Figure 3). Expression data derived from the arrays were validated by qPCR providing independent quantifiable evidence of the magnitude and direction of change of individual genes. The observation that only 768 genes were modulated following *RPL19* knockdown, with the levels of mRNA for a wide range of proteins either maintained or elevated, suggests that ribosomal protein RPL19 is differentially involved in protein synthesis rather than affecting all cellular protein synthesis in a non-specific manner.

Functional enrichment analysis identifying some 20 Gene ontology (GO) biological process terms and three molecular function terms (Supporting Information Table S3) to be significantly associated (*p*<0.001) with the knockdown (*p*<0.001). Additionally 13 KEGG pathways had a significantly over-representation of genes differentially expressed between *RPL19-*PC-3M_clone ST-3_ and PC-3M_scramble_ (Supporting Information Table S4). Ingenuity pathway analysis was used to identify significant biological networks and pathways in which the genes expressed differentially as a consequence of *PRKC-ζ* knockdown were involved. The top five ranked interlinked pathways (Supporting Information Table S5) and the three Gene ontology (GO) molecular function terms (Supporting Information Table S6) are highly significant (*p*≤10^−27^) with respect to genes differentially expressed after *RPL19* knockdown.

#### Ribosomal protein genes

The hypothesis that siRNA-induced down-regulation of *RPL19* might be compensated by modulation of other ribosomal proteins was addressed by assessment of the relative expression of the mitochondrial large ribosomal protein gene sequences (n = 71) and the cytoplasmic large ribosomal protein gene sequences (n = 136) to discover whether up-regulation of a gene already expressed or neoexpression of a previously silent ribosomal protein gene had occurred. Of the latter cohort, 44 genes encoded known RPs, 7 were RP-like and 5 were RP pseudogenes. The number of sequences representing each gene ranged from one (19 genes) to 14 (*RPL21*). RPL19 was identified by a single sequence. According to SCOP (Structural Classification of Proteins, latest release 9^th^ November 2010, http://scop.mrc-lmb.cam.ac.uk/scop) RPL19 is a member of the protein superfamily of translation proteins containing the SH3-like barrel structural domain within the Class comprising all beta proteins. The family also contains ribosomal proteins RPL14e, RPL21e and RPL24p and the C-terminal domain of RPL2 (http://supfam.org/SUPERFAMILY/cgi-bin/scop.cgi?sunid=50104). Alternatively, RPL19 protein could be replaced by RPL29 or RPL39e, being structurally similar members of the α-helical group of globular RPs with extended tails able to bind mRNA [43]. Although fluctuations occurred in the levels of expression of individual RPL gene sequences following *RPL19* knockdown, these were not significant, including that of ribosomal protein gene *RPL23A* also located on chromosome 17q11.2. Only expression of mitochondrial *MRPL42* was significantly down-regulated (*p*<0.05). No enhanced expression of any RP gene was detected. Thus, inhibition of *RPL19* with loss of RPL19 protein was not compensated by a different RP gene. Conversely, the effects of reducing *RPL19* could be mediated by the coding-independent function of the gene or its pseudogene mRNAs [44].

#### Glycosyltransferase genes

Transformation of epithelial cells from a benign to a malignant phenotype is often accompanied by structural changes in the oligosaccharide domains of cellular glycoproteins and glycolipids [45]. Particularly, expression of sialylated and *β-1,6* branched N-linked oligosaccharides are required for cancer cell invasion and metastasis [46]. The key enzyme in this process is mannosyl (*α-1,6*-)-glycoprotein *β-1,6*-N-acetyl-glucosaminyltransferase encoded by gene *MGAT5* and regulated by signaling pathway RAS-RAF-MAPK. Together with *PTEN*, *MGAT5* regulates the membrane dynamics of PI3K/Akt signaling to promote the invasive malignant phenotype [47]. In the event that malignancy is reduced following manipulation of cellular phenotype, changes in cell-surface oligosaccharide structures are postulated to occur. Such changes, mediated by glycosyltransferases may be evidenced by altered expression of the corresponding genes. Of the 768 genes differentially-expressed, only two glycosyltransferase genes were significantly affected following *RPL19* knockdown (Supporting Information Table S7). Unlike the spectrum of glycosyltransferases modulated following si-RNA knockdown of *PRKC-ζ* in PC-3M cells [33], no change was apparent in silayl- or fucosyl-transferase genes. However, a 4-fold reduction was identified in the level of *MGAT4A* (*p*<0.05) that encodes the enzyme mannosyl (*α-1,3-)-*glycoprotein *β-1,4-*N-acetylglucosaminyltransferase and is involved in mediating glycosylation of the proteins encoded by *SLC43A3* (proteoglycan 2), SLC14A1 (urea transporter) and *SLC8A1* (sodium/calcium exchanger), thereby controlling their cell-surface expression. Indeed, all three latter genes were modulated following *RPL19* knockdown. Conversely, a 2∼3-fold increase was identified in the level of *GALNACT-2* (*p*<0.05) that encodes the enzyme chondroitin sulfate N-acetylgalactosaminyltransferase 2 and transfers N-acetylgalactosamine (GalNAc-) from UDP-GalNAc [48] to chondroitin, chondroitin sulfate, preferentially to complex oligosacfocharides containing *β1→4* linkages[49], such as those generated by *MGAT4A*.

#### Ion channels and associated genes

The malignant phenotype of prostatic epithelial cells can be modulated by differential expression of ion channels [50]–[52]. Studies from this laboratory [52] and elsewhere [53] have established a functional relationship between voltage-gated ion channels and the invasive phenotype of prostate cancer cells [50]. Interrogation of the expression arrays revealed several ion channels and some associated genes to be modulated following *RPL19* knockdown (Supporting Information Table S3). Potassium channels showed a mixed response. The voltage-gated K^+^ channel alpha and beta subunits (*KCNQ2* and *KCNAB2*) were down-regulated 3.5- and 2.25-fold, respectively (*p*<0.05 for both). The inward-rectifier K^+^ channels (*KCNJ6* and *KCNJ12*) showed a mixed response, being up-regulated 5.5-fold and down-regulated 2.5-fold, respectively (*p*<0.01 for both). Two voltage-gated Na^+^ channel genes (*SCN3A* and *SCN9A*) were both up-regulated, 9-fold (*p*<0.005) and 2.3-fold (*p*<0.05), respectively. Finally, two voltage-gated Cl^-^ channels**/**Cl^-^-H^+^ antiport transporters (*CLCN4* and *CLCN5*) were both up-regulated, 2.1 and 1.8-fold, respectively (*p*<0.05 for both).

#### Other genes and associated networks

**Figure 4:**
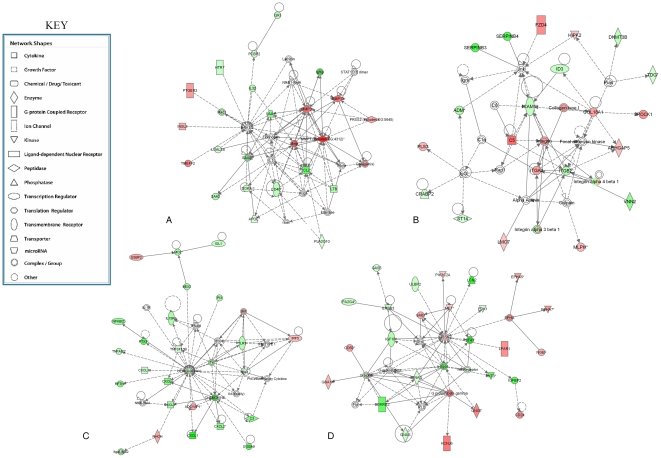
Gene Ontology (GO) enrichment pathway analysis. Analysis of genes modulated following *RPL19* knockdown identified five interlinked pathways principally affected (Supporting Information Table S5). Four of these include genes encoding MMP enzymes (A); the ICAM1-integrin complex (B); the NFκB complex (C) and PI3K regulation (D). This analysis confirmed several genes modulated by down-regulated expression of *RPL19* to be interconnected, emphasizing the numerous pathways for cross-talk between apparently distinct biological processes.

**Figure 5:**
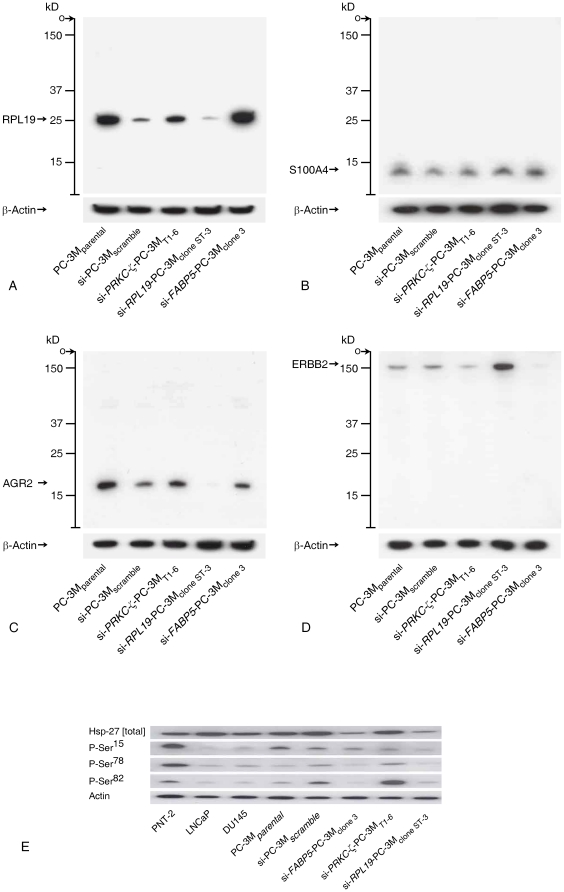
Analysis of protein expression by cells following knockdown of *RPL19*. In these studies, comparison was made with si-*PRKC-ζ*-PC-3M_T1-6_ [33] and si-*FABP5*-PC-3M_clone 3_ [62] RNAi-knockdown cells to confirm that changes in protein levels were specific to *RPL19* knockdown and not part of a general response to gene inhibition using si-RNA. After staining with primary antibodies, membranes were re-stained for beta-actin. The intensity of this band was used to normalize individual protein levels. A. RPL19: Following *RPL19* knockdown, levels reduced to ∼5% of those in the PC-3M_parental_ cells whereas levels were maintained in si-*PRKC-ζ-*PC-3M_T1-6_ and si-*FABP5*-PC-3M_clone 3_ cells. B. S11A4: Levels were maintained in all cell-lines, being unaffected by *RPL19* knockdown. C. AGR2: Expression of protein abrogated following *RPL19* knockdown but maintained in si-*PRKC-ζ*-PC-3M_T1-6_ and si-*FABP5*-PC-3M_clone 3_ cells. Absence of AGR2 defines, in part, the non-malignant phenotype of prostate epithelium [55]. D. ERBB2: Enhanced expression of ERBB2 occurring in the *RPL19* knockdown cells is strong evidence against a single functional amplicon in prostate cancer that contains both *RPL19* and *ERBB2*. In contrast, levels of ERBB2 were reduced in the si-*PRKC -ζ*-PC-3M_T1-6_ cells and undetectable in the si-*FABP5*-PC-3M_clone 3_ cells. E. Differential expression of Hsp-27 in prostate cancer cell-lines, including locus-specific forms of the phosphorylated protein, showing selective loss of the protein following RPL19 knockdown, although not in the si-*PRKC-ζ-*PC-3M_T1-6_ cells. Loss of total Hsp-27 is characteristic of the non-malignant phenotype of prostatic epithelial cells [105].

None of the cell-cycle control genes, including the 31 we previously showed to be associated with a high probability of prostate cancer progression [54] were modulated in their expression following knockdown or *RPL19*. Similarly, none of the genes recognized to mediate apoptosis were modulated in the transfectants. Of the 19 sequences covering the caspase family of apoptosis genes, *CASP1* was down-regulated ∼7-fold (*p*<0.005) following *RPL19* knockdown. The expression of other members of the family was not altered. In support of the array data, Western blotting confirmed that cleaved caspases -3 and -9 were not expressed either in the PC-3M_parental_ or in the si-*RPL19-*PC-3M_clone ST-3_ transfectant cells. These findings support the proposition that altering expression of *RPL19* does not affect either the cell-cycle or the apoptotic pathways. Conversely, the major pathways affected following *RPL19* knockdown involve networks of genes regulating homeostasis and the interaction between the malignant cells and their environment (Figure 4). As an example, expression of the regulator gene *AGR2* we identified to be elevated in prostate cancers of aggressive phenotype [55] was down-regulated ∼11-fold (*p*<0.02) following *RPL19* knockdown. The product of this gene binds to the receptor ErbB3 and is regulated by the forkhead DNA-binding transcriptional regulators Foxa1 and Foxa2. Western blotting confirmed abolition of this protein in the knockdown cells (Figure 5), supporting the array data (Supporting Information Tables S1 & S2). In contrast, HOXB13 encoding a transcription factor belonging to the homeobox gene family that we showed to be a tissue-specific biomarker of benign and malignant prostatic epithelium [56] was elevated ∼3-fold (*p*<0.001) following *RPL19* knockdown.

### Phenotypic gene expression in prostatic malignancy

#### Protein expression

The finding that the rate of cell proliferation did not decline following gene knockdown suggested that global suppression of protein synthesis was unlikely to have occurred despite expression of an individual ribosomal protein being significantly reduced. However, a differential effect was identified with respect to individual proteins (Figure 5), exemplified by AGR2 that was abrogated in the si-*RPL19-*PC-3M_clone ST-3_ cells while expression of ERBB2 was simultaneously enhanced. The observation that individual proteins were differentially affected suggests the biological effects of reducing *RPL19* to be gene-specific and protein-specific rather than a global down-regulation of protein synthesis. The enhanced level of ERBB2 provides additional evidence against a common amplicon in chromosomal region 17q11.2–q12 since expression of *RPL19* and *ERBB2* were divergent (Figure 5).

#### Hsp-27 expression and phosphorylation status

Western blotting confirmed the level of total Hsp-27 protein to be lower in the si-*RPL19*-PC-3M_clone ST-3_ and si-*FABP5*-PC-3M cells than in the PC-3M_parental_, PC-3M_scramble_ and the si-*PRKC-ζ-*PC-3M*_T1-6_* cell-lines (Figure 5). A global reduction in site-specific phosphorylation of si-*RPL19*-PC-3M_clone ST-3_ cells was also identified when compared to PC-3M_parental_ and PC-3M_scramble_, in contrast to the effect of knocking-down *PRKC-ζ* [33].

#### Glycoconjugate expression

Lectin histochemistry, employed to test the hypothesis that *RPL19* knockdown would modulate the profile of sialylated glycoconjugates, revealed no qualitative difference in expression of Neu5Acα2→3Gal- and Neu5Acα2→6Gal- (using *Sambucus nigra* and *Maackia amurensis*, respectively) when the PC-3M_parental_ and si-*RPL19-*PC-3M_clone ST-3_ cells were compared. Staining was abolished in all cell-lines following neuraminidase digestion, confirming specificity of sialic acid expression. Similarly, the lectins from *Ulex europaeus*, *Lotus tetragonolobus* and *Aleuria aurantia* revealed no changes in terminal fucosyl linkages. Thus, suppression of the metastatic phenotype by knockdown of *RPL19* did not involve appreciable loss of sialic acid from the cell surface.

## Discussion

This study provides evidence that posttranscriptional silencing of *RPL19* using RNAi not only abrogates the malignant phenotype of PC-3M prostate cancer cells but is selective with respect to transcription and translation of other genes. In prostate cancer, expression of *RPL19* is significantly elevated, functionally involved in maintaining the malignant phenotype and hence a potential target for therapeutic intervention. Despite its involvement in ribosome structure and function, the data show that the effects of reducing *RPL19* are not global but restricted to a defined cohort of genes and proteins. This observation supports the accumulating evidence of eukaryotic ribosomal specialization in which loss of function phenotypes of individual ribosomal proteins is associated with changes to specific signaling pathways and tissues [57] suggesting that core ribosomal proteins may contribute differentially to translation of distinct subpopulations of mRNAs [58]. Within ribosomes, the role of RPL19 remains undefined. Nevertheless, importance of the gene may be inferred from the number of its paralogous sequences maintained within the eukaryotic genome [59] and the finding that expression of *RPL19* is one of the most stable and consistent genes within the human genome [60], [61]

Analysis of the 768 genes modulated following *RPL19* knockdown revealed a genetic profile distinct from that obtained after siRNA reduction of *PRKCZ* [33] or *FABP5* [62] in the same PC-3M cells. The cohort of modulated genes did not contain any cell cycle-associated genes [54], DNA-binding genes (e.g. *RAD51*) or transcriptional activation genes (e.g. Id-1) we have already reported in aggressive primary prostate cancers [63], [64]. Furthermore, the affected gene-networks did not involve cell adhesion genes or other ribosomal protein genes identified in metastatic breast cancer [65]. However, *AGR2* was down-regulated (>10-fold, *p*<0.001) in the knockdown cells, consistent with our previous finding in non-malignant prostatic epithelium [55]. Although the levels of some ion channel and glycosyl transferase genes were appreciably modulated, individual members of these cohorts were different from those identified following RNAi gene-knockdown of *PRKCZ* [33] providing additional evidence that gene expression is heterogeneous within the benign phenotype.

While enhanced expression of some RP genes has been reported in other human malignancies [66], including lung [28], colorectal [67], prostate [68] and *RPL19* in breast cancer [69] this is the first report to define a functional role for *RPL19* in the malignant phenotype. Although RPL19 protein is an integral component of the large 60S subunit of eukaryotic ribosomes [59], [70] its ribosomal function has not been defined. Nevertheless, reduction in its expression sufficient to modulate the behavioral phenotype from malignant to benign did not involve a detectable alteration in cell proliferation or apoptosis indicating that the phenotypic effects were not simply due to the target cells becoming compromised either metabolically or by diminished protein synthesis. In the absence of non-specific global effects, the data indicate that reducing *RPL19* expression affects discrete populations of genes and proteins, thus shifting the balance of gene expression from a malignant to a benign phenotype.

Intuitively, loss of RPL19 protein might be expected to cause a general decline in ribosome biosynthesis with compromised functionality and commensurate loss in protein synthesis. Under such circumstances, cell proliferation would have decreased without specific effects on particular cellular functions. In contrast, the data indicate the effects to be selective with respect to cell adhesion, stromal invasion and tumorigenesis. Although the role of individual RPs in determining the cellular phenotype of eukaryotic cells remains unclear, current evidence reveals mutations within individual ribosomal proteins to be associated with specific changes in cellular phenotype [71], [72] rather than a general down-regulation of protein synthetic activity. Examples emerging within other fields of protein biology indicate that alternative genes may be recruited to replace defective or deficient proteins [73]–[75]. Although such mechanisms would be important to maintain structure-function relationships within complex organelles, no such examples have been reported to compensate for deficient ribosomal proteins. Subsequent to the loss or replacement of an individual ribosomal protein, the functional activity of the modified organelles would not be identical to that of the original structure, thus providing a drive towards adaptation and evolution of a novel phenotype [76], [77] In addition to protein biosynthesis, many RPs also fulfill extra-ribosomal functions, particularly regulating the quality of gene expression through coupling transcription mechanisms with the processing and transportation of mRNAs [78], [79]. Such effects are stochastic and cannot be predicted because of the complexity gene interactions [80]. Nevertheless, mathematical models are emerging to analyze the effects of insertions and deletions in protein-protein interaction networks and the global changes consequentially induced in cellular structure and function [81]–[83]. Despite protein synthesis being a general function of ribosomes, the precise function of each ribosome depends upon its complement of ribosomal proteins, ribosomal RNAs (rRNAs) and a range of ribosome-associated proteins (PARs) [84]. Ribosome biogenesis is complex and highly regulated [85], [86]. Continuity of the cell-cycle depends upon fidelity of ribosome biogenesis and ceases if ribosome biogenesis becomes impaired [10], [87]–[89], leading to a variety of ribosomopathies [90]. Such data provide evidence that structurally-defective ribosomal components (rRNAs, RPs or PARs) cause disruption of a cell's translational apparatus [91], resulting in alterations to cellular phenotype [92] with the consequence that small changes in molecular structure may cause significant alterations in ribosomal function.

Herein we confirm a functional role for *RPL19* in promoting the malignant phenotype of human prostate cancer cells. Despite significant reduction in the levels of RPL19 mRNA and protein, the finding that cell proliferation was not demonstrably affected challenges the supposition that RPL19 protein is essential for ribosomal structure and/or function and suggests a level of adaptation within ribosomal protein function that enables global protein synthesis to be maintained despite loss of a core ribosomal component. If RPL19 is not a critical component of ribosome structure and/or function, its importance to the malignant phenotype may be related to its extra-ribosomal activities, with the implication that there is no necessity for protein substitution or adaptation at the ribosomal level. Our finding that the patterns of genes and their associated networks modulated by *RPL19* knockdown are distinct from the patterns following *PRKCZ* knockdown in identical cells is consistent with two propositions: First, that loss of individual ribosomal proteins is associated with specific alterations in cellular phenotype. Second, that the non-malignant phenotype is not defined by a single immutable pattern of gene expression but is in flux[93] in the same manner that the patterns of genes expressed in malignant cells are heterogeneous [94], [95]. The possibility of flux between metastable gene networks raises the exciting possibility of therapeutically stabilizing a benign phenotype generated by modulating expression of a key gene and hence constraining a malignant phenotype while leaving non-malignant genomes unaffected.

## Materials and Methods

### Cell lines

Human prostate cell-lines PNT2 (benign) and PC-3M_parental_ (highly malignant) are identical to those described previously [33]. PNT2 cells are non-malignant, androgen-independent and derived from SV-40 immortalization of normal prostatic epithelial cells [96]. PC-3M cells are malignant, also androgen-independent and derived from the bone marrow metastasis of a 62 year-old man [97]. These cells exhibit a high incidence of tumorgenicity and metastasis when xenografted into nude mice [98]. Both cell-lines are histogenically the closest currently available having contrasting behavioral phenotypes and hence the most appropriate as comparators. Gene knockdown derivatives of the PC-3M cell-line si-*FABP5*-PC-3M_clone 3_ [62] and si-*PRKCZ*-PC-3M_T1-6_ [33] described in comparable studies and were employed to reveal similarities and differences in gene-expression following abrogation of the malignant phenotype in PC-3M cells using an identical technique. All cell-lines were grown as monolayer cultures in RPMI 1640 (Invitrogen, Paisley, UK) supplemented with 10% (v/v) fetal calf serum (FCS, Invitrogen), penicillin (1000units/ml), streptomycin (100 µg/ml), and L-glutamine (2 mM). Media for the culture of all subsequent transfected cell-lines were also supplemented with 1 µg/µl Geneticin (Sigma).

### siRNA Knockdown of RPL19 in PC-3M cells

#### Transient transfection

Transient transfections were performed by the reverse transfection technique using siPORT NeoFX Transfection Agent (Ambion, Warrington, UK). Three sequences were initially assessed as potential targets for stable transfection to silence variant “c”, the NM version of the *RPL19* gene (NM_000981). All three sequences were potentially capable of silencing seven of the alternative eight splice variants (a, b, c, d, e, f, g, h) of *RPL19*. The alternative 168 bp variant “i” was incomplete since it did not contain the target. The transfection targets, listed in Table 2, were BLAST-searched and showed homology and similarity only to *RPL19*. Sequences to targets were designed using Ambion's online target design algorithm and purchased from Ambion was also included, pre-annealed at a concentration of 20 nmol.

A negative control siRNA was also included that comprised a nucleotide sequence similar in composition to that of the siRNA but not homologous to any known gene of interest and purchased pre-designed from Ambion. This “scramble” sequence was used to discount non-specific changes in gene expression profiles due to siRNA delivery. Preliminary experiments optimized transient transfection conditions for PC-3M cell-lines. Reverse transfection was performed in a 96 well plate format. Cells were seeded at a density of 8×10^3^ cells/well. The short strand RNA (ssRNA) oligonucleotide sequences were then diluted in a reduced serum medium (OPTI-MEM 1; Invitrogen, Paisley, UK) to a final concentration of 30 nM. This was then overlaid onto the cells that were then incubated at 37°C in 100% humidity in 5% CO_2_/air for 24 hours. Transfection of the RNA oligonucleotide sequences into the cells occurred spontaneously as the cells adhered to their substrata.

#### Stable transfection

After transient transfection had identified Target #1 as the most successful to silence *RPL19* expression, this sequence was used to generate a hairpin siRNA. The following oligonucleotides were purchased from Ambion:

*Top Strand:*

5′-GATCCGCTCATCAAAGATGGGCTGTTCAAGAGACAGCCCATCTTTGATGAGCTTA-3′

*Bottom Strand:*

5′-AGCTTAAGCTCATCAAAGATGGGCTGTCTCTTGAACAGCCCATCTTTGATGAGCG-3′

The default Ambion loop sequence, TTCAAGAGA, was used to complete the hairpin structure. The siRNA expression vector kit used was p*Silencer*™ 4.1-CMV neo (Ambion). Top and bottom strands of the siRNA hairpin oligonucleotide were diluted to 1 µg/ µl in TE buffer and annealed in 50 µl solution according to the manufacturer's instructions. The annealed siRNA template was ligated into the p*Silencer* 4.1-CMV vector using T4 DNA ligase (5 U/µl) and the products cloned into DH5α cells (Invitrogen). Transformed cells were grown for 16 hours on LB plates containing 100 µg/ml ampicillin at 37°C. A negative control of non-transformed competent cells was also included. Clones were picked and the DNA plasmid isolated using a Qiaprep spin Miniprep Kit (Qiagen, Crawley, UK). Isolated plasmids were digested with *Bam*HI and *Hin*dIII (New England Biolabs, Hitchin, UK) and the presence of the siRNA 55 bp insert was confirmed by sequencing prior to the siRNA expression vector being used to transfect recipient prostate cancer cell-lines. Orientation of the insert was confirmed by DNA sequencing (Lark Technologies, Essex, UK) using internal sequence primers.

#### Transfection of siRNA RPL19 silencing construct and control

1.5×10^5^ PC-3M_parental_ cells were transfected with p*Silencer* 4.1 CMV *RPL19* siRNA (1 µg) using *SiPORT XP-1* (3 µl) reagent (Ambion, Warrington, UK) in 6-well-plates (35 mm diameter). 24 hours after transfection, 500 ng/ml of G418 was added to medium RPMI1640 for selection. After 9-10 days selection, individual colonies from single cells containing stable clones were isolated using ring cloning and transferred into 24-well plates with medium containing G418 at 500 ng/ml. Simultaneously, 1.5×10^5^ PC-3M_parental_ cells were transfected with p*Silencer* 4.1 CMV-scramble-insert (1 µg). Thereafter, these cells were cultured, cloned and employed as the controls to assess changes in expression of genes and proteins by the knockdown cells.

#### RNA extraction and cDNA synthesis

Total RNA was extracted with RNeasy Mini Kits (Qiagen). Total RNA concentration was measured using a NanoDrop instrument (Labtech, Ringmer, UK) and RNA integrity assessed with a 2100 Bioanalyser (Agilent, Santa Clare, USA). The RNA integrity number (RIN) for all RNA used exceeded 9.0. First strand cDNA was synthesized from 0.5 µg total RNA using AffinityScript™ Multiple Temperature cDNA synthesis kits (Stratagene, La Jolla, USA) according to the manufacturer's protocol.

#### Quantitative Real-Time PCR (qPCR)

*RPL19* mRNA expression levels were quantified by qPCR and normalized relative to human β-actin mRNA expression. An MX3305P Real Time PCR machine (Stratagene) was used for all reactions. Reaction volumes were in 25 µl comprising 12.5 µl Stratagene's Brilliant® SYBR® Green Master Mix (2X), 0.5 µM of both forward and reverse primers and 1 µl cDNA and 11.5 µl water. Primers for qPCR were designed to span exon/exon boundaries within the mRNA to avoid amplification of genomic DNA. Primers designed for *RPL19* and human β-actin are listed in Table 1. Both primers were optimised at 60°C. Cycling conditions for the reaction were: 95°C for 15 minutes, then 40 cycles at 94°C for 15 seconds, 63°C for 30 seconds, plate read and 72°C for 30 seconds with a final extension at 72°C for 10 minutes. Melting curves were generated to detect primer-dimer formation and to confirm gene-specific peaks for targets.

#### Growth characteristics and invasiveness of si-*RPL19* cells *in-vitro*

An assay was established to identify the effect of *RPL19* suppression on cellular proliferation. The relative growth rates of PNT2, PC-3M_parental_, PC-3M_scramble_ and si-*RPL19-*PC-3M_clone ST-3_ transfectant cells were measured by proliferation assay. Exponentially-growing cells were seeded in triplicate sets at a density of 1×10^3^ cells/ml/well in 24-well plates. Over 10 days at 24–48 hour intervals, cell proliferation was calculated by measuring the increase in cell numbers in each replicate using a conventional MTT assay [99]. Apoptosis was quantified using flow cytometry. Cells from PNT2, PC-3M_parental_, PC-3M_scramble_ and two si-*RPL19*-PC-3M clones were seeded at 2×10^5^ cells/ml in 75 cm^2^ tissue culture flasks and the assay started prior to cells reaching confluence. Duplicate flasks were established in which cells were exposed to 4 µM camptothecin (Sigma-Aldrich) dissolved in DMSO for 24 hours before harvesting. Camptothecin, a potent inhibitor of topoisomerase I, induces apoptosis in a dose-dependent manner *in-vitro* [100], [101]. Cells were harvested by trypsinization, washed twice with PBS and re-suspended in buffer from the BioVision Annexin V-FITC kit in a 5 ml flow cytometry tube. AnnexinV-FITC (5 µl) and propidium iodide (10 ng in 5 µl aqueous solution) were added and the tubes incubated for 10 minutes in darkness at 4°C. Quantitative analyses of apoptotic cell levels were performed using an Epics Flow Cytometer (Beckman Coulter). The procedure was performed three times using biological replicates. Invasiveness of the si-*RPL19* transfectants was assessed *in-vitro* [52]. At 24-hour intervals, following fixation and staining with Crystal Violet (Sigma-Aldrich, St Louis, USA), invasion was measured by counting the number of cells transmigrating the membrane to its under-surface [33].

#### Tumorigenicity and *RPL19* protein expression *in-vivo*

All studies were performed under the conditions of UK Home Office Project License PPL 40/2270 [33]. Tumorigenicity was assessed by injecting cells (2×10^6^ cells in 0.2 ml PBS) into a single subcutaneous site in the right shoulder of 8 week old male Nu/nu mice (Harlan Ltd., Oxon, UK). Four groups of cells were assessed: PC-3M_control_, PC-3M_scramble_ and si-*RPL19*-PC-3M clones -#1 and -#2. Of these two, si-*RPL19*-PC-3M_clone ST-3_ exhibited the most pronounced suppression of *RPL19* and was used in the microarray and invasion assay experiments. Clone si-*RPL19*-PC-3M_#2_ exhibited suppression mid-way between that of si-*RPL19*-PC-3M-_#1_ and si-*RPL19*-PC-3M_scramble_. Tumor growth was monitored twice-weekly by measuring the largest (a) and smallest (b) superficial diameters. Tumor volume (V) was then calculated using the formula V = a x b^2^/2 [102]. When any tumor reached the maximum size allowed under the conditions of the Home Office Project Licence PPL 40/2270, all mice were sacrificed. Each animal was submitted to autopsy to identify appearance of metastatic tumor nodules. Subcutaneous primary tumors together with heart, liver and lungs were removed and weighed. All tissues were processed and embedded in paraffin wax. Histological sections cut at 4 µm and stained with Gill’s hematoxylin for microscopic examination.

Expression of RPL19 protein in human prostate epithelial cells grown as xenografts in nude mice was detected using a mouse monoclonal antibody (Abnova, Taiwan; #H0000 6143-MO1) diluted to 1∶1000 in REAL antibody dilutent (Dako, cat. no. S2022). Prior to staining, antigen retrieval employed PT-Link with EnVision FLEX, high pH target retrieval solution. Staining was performed on a Dako Autostainer using a labeled polymer-HRP detection system (Dako, EnVision FLEX, K8000). Immunostained sections were counterstained with hematoxylin, dehydrated and mounted. Negative controls comprised duplicate tissue sections processed identically but with replacement of the primary antibody by a 1% (w/v) solution of bovine serum albumin. Specimens were considered positive only when at least 5% of the epithelial cells (either normal or malignant) unequivocally expressed RPL19 staining [103]. This cut-off was the same as that used to distinguish positive and negative immunohistochemical staining in our previous studies [104], [105]. Staining was assessed as negative, weakly positive or only focally positive (low-level expression), or strongly positive (high-level expression) and scored as 0, 1, 2 or 3, respectively.

### Microarray analysis

#### Microarray validation

Gene expression profiles were validated in the knockdown cells using qPCR to confirm the expression level of *NFKB1A*, *TNFSR6*, *MMP3* and *MMP10* (Supporting Information Figure S1) in addition to *PLAT*, *HSPB1, CDKN2C* and *FOXA2*, previously employed to validate these arrays [33] when normalized against human β-actin. The primers and amplicon sizes are listed in Table 1. All annealing temperatures were 60°C and cycling conditions as described previously [33].

#### Gene microarray and expression analysis

The effect of suppressing *RPL19* by gene knockdown on whole genome expression profiles was investigated using two-color Agilent Human genome 44k microarrays. Each hybridization was a distinct biological replicate. The design incorporated five cell-lines treated as fixed biological factors: PNT-2, PC-3M_parental_, si-PC-3M_scramble_, PC-3M_pool_ and si-*RPL19-*PC-3M_clone ST-3_ (*RPL19* knockdown). The si-PC-3M_scramble_ cell-line was employed as the common control comparator to identify differences in gene expression between these cells and all other cell-types. Hybridizations and data acquisition were performed according to the Agilent Human Genome Microarray (MA) 44K protocol. Spatial representations of the hybridization signals were examined to confirm absence of technical artifacts. The distribution of background and foreground signals and pre-normalization MA plots were examined to measure the quality of the hybridization. Low quality spots identified by the Agilent image processing software were not used in the subsequent analyses. Expression signal estimates were derived from the red (Cy3) and green (Cy5) Agilent Processed Signal data by normalizing using the LOESS algorithm and background correction using a fitted convolution of normal and exponential distributions [106], [107]. Expression analysis of log_2_ transformed normalized data was performed in the R statistical programming language (R v 2.10.0) using the BioConductor framework [108]. Gene expression was modeled with a fixed effects linear model using BioConductor limma [109]. Various contrasts were examined such as “PNT *vs* scramble” and “knockdown *vs* scramble”. For each contrast, a moderated *t*-statistic was computed for each probe with the resulting *p*-values adjusted for multiple testing using Benjamini and Hochberg's method to control the false discovery rate [110]. This is the same as an ordinary *t*-statistic except that the standard errors have been moderated across genes using a Bayesian model. Those sequences with an adjusted *p*-value<0.05 were considered significantly differentially expressed between the two groups being compared. GO terms and KEGG networks that were significantly associated with the genes expressed differentially between si-*RPL19-*PC-3M_clone ST-3_ and PC-3M_parental_ cell lines were assessed using hypergeometric tests (*p*<0.001) [111]. The list of genes expressed differentially between si-*RPL19-*PC-3M_clone ST-3_ and PC-3M_parental_ cell lines was uploaded into the Ingenuity pathway analysis application (Ingenuity® Systems, www.ingenuity.com). A score was computed for each network according to the fit of the original set of significant genes. This score reflects the negative logarithm of the *p*-value, which indicates the likelihood of the focus genes in a network being found together as a result of random chance. To be considered significant, the adjusted *p* value of the differences between the level of expression in the two cell lineages were ≤0.01. Genes were grouped according to whether they were significantly up-regulated (Supporting Information Table S1) or significantly down-regulated (Supporting Information Table S2) and thereafter according to function. The obtained gene expression data are MIAME-compliant and have been deposited with the NCBI GEO database.

### Phenotypic gene expression in prostatic malignancy

#### Protein expression

To analyse the effect of reducing *RPL19* expression on the protein-synthetic function of ribosomes, Western blotting was performed using a range of commercially-available antibodies (Supporting Information Table S8). Proteins were extracted from ∼1×10^7^ cells from each line. Cell pellets were suspended in 1 ml of CelLytic-M lysis buffer (Sigma C2978) containing 10 µl protease inhibitor cocktail (Sigma P8340), 10 µl PMSF (0.1 mg/ml), Na_3_VO_4_ (1 mM) and NaF (1 mM). Protein concentrations were determined by Bradford assay (BioRad kit 500-0006). Aliquots containing ∼10 µg cell lysate proteins were separated electrophoretically at 150 V in 12.5% (w/v) polyacrylamide NextGel quick-cast separating gels (Amresco, Solon, OH). Separated proteins were transferred onto PVDF membranes (GE Healthcare, RPN303F), at 100 V for 1 hour, blocked with a suspension of powdered dried milk in PBS (100 mM, pH 7.6) before incubation at 4°C with primary antibodies. After washing and incubation with the corresponding anti-(mouse Ig)- or anti-(rabbit Ig)-HRP antiserum at 1∶10,000 dilution for 1 hour, washing and incubation in ECL Plus reagent (GE Healthcare, RPN 2133) for 5 minutes, exposure to Amersham Hyperfilm (GE Healthcare, 28906839) for 5 seconds before being developed and fixed. To quantify protein expression, membranes were re-incubated with an anti-beta actin mouse monoclonal antibody for 30 minutes. Bound anti-actin antibody was detected as described. A strong single band at 42 kDa was observed in all cases.

#### Hsp-27 expression and phosphorylation status

Hsp-27 is an independent biomarker of the aggressive malignant phenotype of human prostate cancer [105]. Although no functional relationship has been reported between Hsp-27 and PKC-ζ, it was hypothesized that amelioration of malignancy following *RPL19* knockdown would be accompanied by a reduction in the level of Hsp-27 expression. Expression of Hsp-27 is a validated biomarker of prostate cancer malignancy [105], [112]. Therefore, Western blotting was performed on the proteins extracted from ∼1×10^7^ cells from each line to identify total Hsp-27 as well as the differential phosphorylation of this protein at Ser^15^, Ser^78^ and Ser^82^. All methodologies used were identical to those previously reported [33].

#### Glycoconjugate expression

The behavior of malignant epithelial cells is influenced by expression of cell-surface complex glycoconjugates, particularly sialic acids [46], [113]–[115]. To assess the potential effects of *RPL19* knockdown on cell-surface oligosaccharide expression, cell-blocks were prepared from cell-lines PNT2, PC-3M_parental_, PC-3M_scramble_, and si-*RPL19-*PC-3M_clone ST-3_. Cell pellets were processed and embedded in paraffin wax blocks [33], [116]. Sections were cut at 5 µm and stained for Neu5Acα2→3Gal- and Neu5Acα2→6Gal- using biotinylated lectins (Vector Laboratories, Peterborough, UK) from *Sambucus nigra* and *Maackia amurensis* respectively [117], [118]. The biotinylated lectins from *Ulex europaeus*, *Lotus tetragonolobus* and *Aleuria aurantia* were employed to detect terminal fucosyl linkages. Negative controls included the absence of staining when the lectins were not included in the staining protocol and the abolition of staining following pre-treatment of the slides with neuraminidase prior to incubation with the lectins [119]. Lectin-binding was detected using an avidin-peroxidase conjugate visualized following polymerization of 3-3′ diaminobenzidine (DAB).

### Supporting Information Material

This information contains Supporting Information Figure S1 confirming the validation of the arrays by PCR and additional data-tables providing detailed information on the alterations in gene expression, including their involved networks, induced following knockdown of *RPL-19*. This material supports, but does not extend, the findings and conclusions of this study.

## Supporting Information

Figure S1 As well as the gene-sequences employed previously [33] four additional sequences were used to interrogate genes up-regulated and down-regulated and thus validate the levels of expression detected by array-analysis. As with the arrays, the levels were quantified relative to PC-3M_scramble_ cells that were set at unity.
(TiFF)

Table S1 **Top 50 genes up-regulated (fold change).** Genes are arranged in descending order according to log_2_ fold change with corresponding *p*-values.
(DOCX)

Table S2 **Top 50 genes down-regulated (fold change).** Genes are arranged in descending order according to log_2_ fold change with corresponding *p*-values.
(DOCX)

Table S3 **Gene ontology terms.** Gene ontology (GO) biological process terms found to be significantly associated with genes significantly differentially expressed after knockdown of RPL19 using hypergeometric tests.
(DOCX)

Table S4 **KEGG pathways.** KEGG pathways containing genes significantly differentially expressed after *RPL19* knockdown using hypergeometric tests.
(DOCX)

Table S5 **Pathways modulated after** ***RPL19*** **knockdown.** Top five interlinked pathways containing genes significantly differentially expressed after *RPL19* knockdown using hypergeometric tests.
(DOCX)

Table S6 **Gene ontology molecular function terms.** Gene ontology (GO) molecular function terms significantly associated with genes differentially expressed after knockdown of *RPL19* using hypergeometric tests.
(DOCX)

Table S7 **Glycosyltransferase and ion-channel genes modulated following** ***RPL19*** **knockdown.** Following *RPL19* knockdown, modulated expression of only two glycosyltransferase genes was detected but with more profound changes to ion channels indicating significant changes to cellular homeostasis.
(DOCX)

Table S8 **Characteristics of antibodies used to analyze changes in proteins expressed following** ***RPL19*** **knockdown.** Details of protein expression by Western Blotting analysed using a range of mono-specific antibodies to define changes in cellular phenotype following *RPL19* knockdown.
(DOCX)
