# Supplementary figures and images for: siRNA Knockdown of Ribosomal Protein Gene RPL19 Abrogates the Aggressive Phenotype of Human Prostate Cancer
Source: PLoS One. 2011 Jul 22;6(7):e22672. doi: 10.1371/journal.pone.0022672 (PMC3142177; doi:10.1371/journal.pone.0022672)

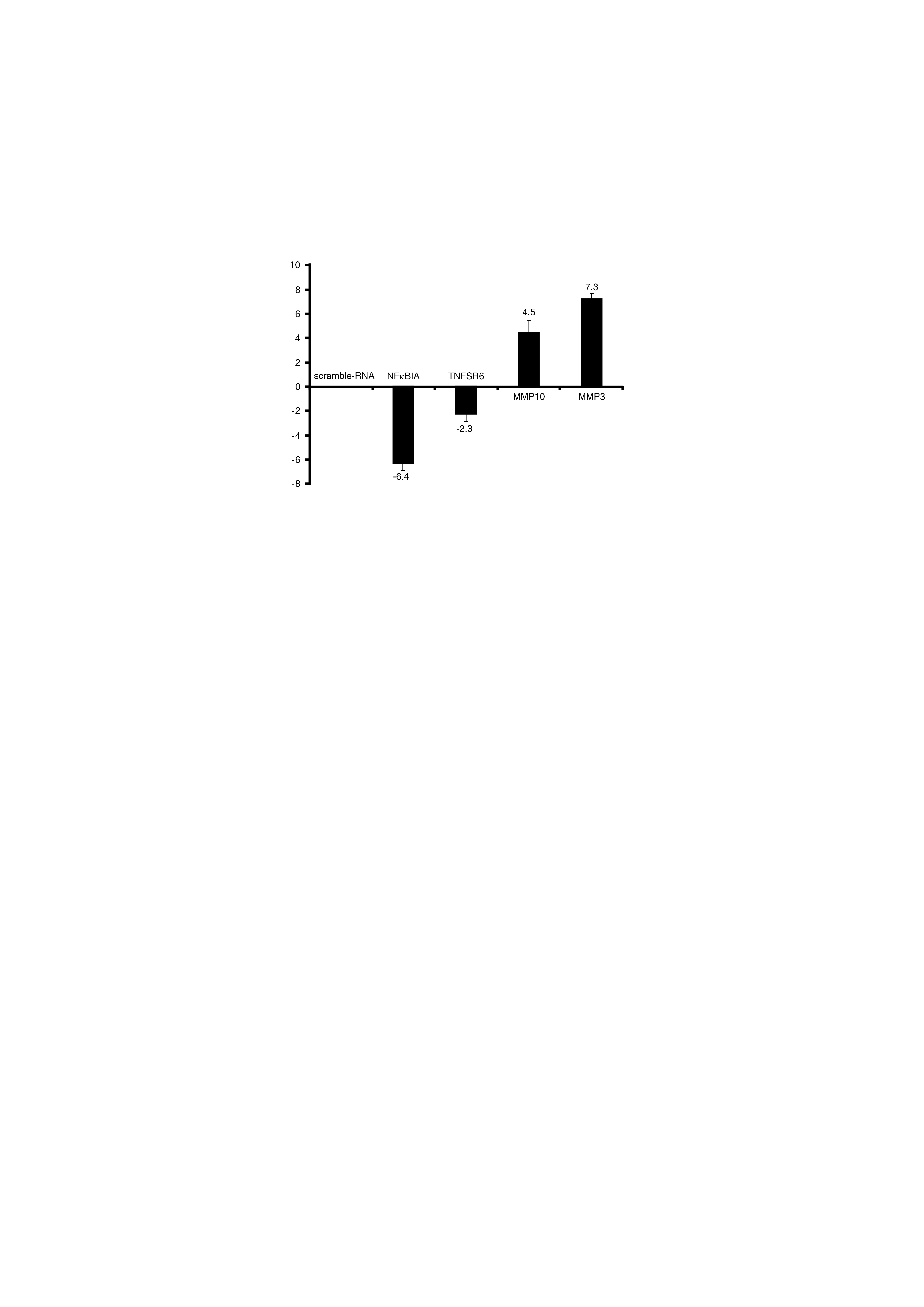

Supplement: Figure S1 — As well as the gene-sequences employed previously [33] four additional sequences were used to interrogate genes up-regulated and down-regulated and thus validate the levels of expression detected by array-analysis. As with the arrays, the levels were quantified relative to PC-3Mscramble cells that were set at unity. (TiFF) [file pone.0022672.s001.tiff]
