# Supplementary material for: siRNA Knockdown of Ribosomal Protein Gene RPL19 Abrogates the Aggressive Phenotype of Human Prostate Cancer
Source: PLoS One. 2011 Jul 22;6(7):e22672. doi: 10.1371/journal.pone.0022672 (PMC3142177; doi:10.1371/journal.pone.0022672)
Supplement: Table S1 — Top 50 genes up-regulated (fold change). Genes are arranged in descending order according to log2 fold change with corresponding p-values. (DOCX) [file pone.0022672.s002.docx]

**Supporting Information Table S1 - Top 50 genes up-regulated (fold-change)**

| **Gene Symbol** | **Gene Name** | **Systematic Nomenclature** | **log_2_ Fold Change** | **Adjusted *p*-Value** |
| --- | --- | --- | --- | --- |
| FSTL5 | Follistatin-like 5 | NM_020116 | 5.52 | 0.0005 |
| C3orf14 | Chromosome 3, open reading frame 14 | NM_020685 | 4.41 | 0.0133 |
| TRBV5-4 | T cell receptor beta variable 5-4 | BC028083 | 4.23 | 0.0122 |
| MMP1 | Interstitial collagenase | NM_002421 | 3.78 | 0.0042 |
| MAP1B | Complex locus encoding microtubule-associated protein 1B. | NM_005909 | 3.69 | 0.0236 |
| MAGEB2 | Melanoma antigen family B, 2 | NM_002364 | 3.65 | 0.0010 |
| GAGE7 | G antigen 7 | NM_021123 | 3.33 | 0.0123 |
| SCN3A | Sodium channel, voltage-gated, Type III α | NM_006922 | 3.20 | 0.0028 |
| AHRR | Aryl hydrocarbon receptor repressor | NM_020731 | 3.05 | 0.0140 |
| LXN | Latexin | NM_020169 | 3.00 | 0.0042 |
| GAGE3 | G antigen 3 | U19144 | 2.96 | 0.0042 |
| SAMD3 | Sterile alpha motif domain-containing 3 | NM_001017373 | 2.89 | 0.0165 |
| TTN | Complex locus encoding titin and hypothetical protein FLJ39502. | NM_133378 | 2.87 | 0.0027 |
| PCDHB2 | Protocadherin β2 | NM_018936 | 2.86 | 0.0070 |
| TNXB | Tenascin XB | NM_032470 | 2.84 | 0.0005 |
| AK055647 | Hypothetical cytoplasmic protein FLJ31085 | AK055647 | 2.80 | 0.0160 |
| C5 | Complement component 5 | NM_001735 | 2.68 | 0.0202 |
| GRPR | Gastrin-releasing peptide receptor | NM_005314 | 2.67 | 0.0207 |
| AL355687 | Hypothetical protein zoytaw | AL355687 | 2.65 | 0.0367 |
| MMP3 | Stromelysin 1 | NM_002422 | 2.59 | 0.0264 |
| EN1 | Engrailed homeobox 1 | NM_001426 | 2.59 | 0.0022 |
| CR613436 | Hypothetical protein lawfeeby | CR613436 | 2.57 | 0.0236 |
| PMEPA1 | Prostate transmembrane protein, androgen induced , variant 1 | NM_020182 | 2.55 | 0.0183 |
| MMP13 | Collagenase-3 | NM_002427 | 2.52 | 0.0088 |
| FN1 | Fibronectin 1 | NM_212482 | 2.49 | 0.0295 |
| LRRC34 | Leucine rich repeat containing 34 | NM_153353 | 2.42 | 0.0062 |
| KCNJ6 | Potassium inwardly-rectifying channel, subfamily J, member 6. | NM_002240 | 2.42 | 0.0078 |
| AK055302 | Hypothetical protein tini | AK055302 | 2.32 | 0.0311 |
| EPHA5 | EPH receptor A5 | BX537946 | 2.31 | 0.0239 |
| LUZP2 | Leucine zipper protein 2 | NM_001009909 | 2.27 | 0.0398 |
| SLC14A1 | Solute carrier family 14, member 1 | NM_015865 | 2.27 | 0.0341 |
| SLITRK6 | SLIT and NTRK-like family, member 6 | NM_032229 | 2.26 | 0.0155 |
| C6orf32 | Chromosome 6, open reading frame 32 | AB002384 | 2.26 | 0.0042 |
| C14orf78 | Chromosome 14, open reading frame 78 | BC090889 | 2.25 | 0.0351 |
| LOC572558 | Phosphoglucomutase-like - 5 | AY343891 | 2.23 | 0.0350 |
| AK091573 | Limbic system associated membrane protein | AK091573 | 2.23 | 0.0097 |
| ZNF248 | Zinc finger protein 248 | NM_021045 | 2.22 | 0.0264 |
| TMEFF2 | Transmembrane protein with EGF- and follistatin-like domains 2 | NM_016192 | 2.22 | 0.0482 |
| GNG7 | G protein, gamma 7 | NM_052847 | 2.21 | 0.0118 |
| CCDC50 | Coiled coil domain containing 50 | NM_178335 | 2.21 | 0.0158 |
| GREM2 | Gremlin 2 | NM_022469 | 2.20 | 0.0429 |
| DKK1 | Dickkopf homolog 1 and hypothetical protein LOC729054 | NM_012242 | 2.19 | 0.0394 |
| BC031314 | Hypothetical protein garpo | BC031314 | 2.17 | 0.0133 |
| HMCN1 | Hemicentin 1 | NM_031935 | 2.17 | 0.0042 |
| C14orf37 | Chromosome 14, open reading frame 37 | NM_001001872 | 2.14 | 0.0213 |
| CR610949 | Hypothetical protein vawgo | CR610949 | 2.13 | 0.0091 |
| APC2 | Adenomatosis polyposis coli 2 | NM_005883 | 2.13 | 0.0043 |
| LRRC6 | Leucine rich repeat containing 6 | NM_012472 | 2.12 | 0.0062 |
| SLC8A1 | Solute carrier family 8, member 1 | NM_021097 | 2.12 | 0.0212 |
| IFTZ74 | Intraflagellar transport 74 homolog | NM_025103 | 2.11 | 0.0042 |
