# Supplementary material for: siRNA Knockdown of Ribosomal Protein Gene RPL19 Abrogates the Aggressive Phenotype of Human Prostate Cancer
Source: PLoS One. 2011 Jul 22;6(7):e22672. doi: 10.1371/journal.pone.0022672 (PMC3142177; doi:10.1371/journal.pone.0022672)
Supplement: Table S2 — Top 50 genes down-regulated (fold change). Genes are arranged in descending order according to log2 fold change with corresponding p-values. (DOCX) [file pone.0022672.s003.docx]

**Supporting Information Table S2 - Top 50 genes down-regulated (fold-change)**

| **Gene Symbol** | **Gene Name** | **Systematic Nomenclature** | **log_2_ Fold Change** | **Adjusted *p*-Value** |
| --- | --- | --- | --- | --- |
| GPC4 | Glypican 4 | NM_001448 | -2.88 | 0.0005 |
| FAM26B | Complex locus family with sequence similarity 26, member B. | NM_015916 | -2.90 | 0.0062 |
| SLPI | Leukocyte peptidase inhibitor | NM_003064 | -2.95 | 0.0062 |
| C3 | Complement component 3 | NM_000064 | -3.01 | 0.0018 |
| SAA1 | Serum amyloid A1 | NM_000331 | -3.05 | 0.0147 |
| HOXB6 | Homeobox B6 | NM_018952 | -3.05 | 0.0038 |
| MAGEC2 | Melanoma antigen family C, 2. | NM_016249 | -3.06 | 0.0484 |
| RFTN1 | Raftin, lipid raft linker1 | NM_015150 | -3.07 | 0.0015 |
| CXCL6 | Chemokine ligand 6 | NM_002993 | -3.07 | 0.0482 |
| PDZD2 | PDZ domain containing 2 | NM_178140 | -3.11 | 0.0017 |
| ZC3H12A | Zinc finger CCCH-type containing 12A. | NM_025079 | -3.11 | 0.0010 |
| TRIM15 | Tripartite motif-containing 15 | NM_033229 | -3.11 | 0.0140 |
| C15orf48 | Chromosome 15, open reading frame 48 | NM_032413 | -3.18 | 0.0009 |
| SLC43A3 | Solute carrier 43 member 3 | NM_199329 | -3.24 | 0.0002 |
| IGSF3 | Immunoglobulin superfamily, member 3. | NM_001542 | -3.25 | 0.0013 |
| MBNL3 | Muscleblind-like 3 | NM_018388 | -3.30 | 0.0206 |
| TMLHE | Trimethyllysine hydroxylase, epsilon | NM_018196 | -3.44 | 0.0005 |
| HOXB9 | Homeobox B9 | NM_024017 | -3.49 | 0.0123 |
| AGR2 | Anterior gradient 2 homolog | NM_006408 | -3.53 | 0.0153 |
| PCSK9 | Proprotein convertase subtilisin/kexin type 9 | NM_174936 | -3.55 | 0.0147 |
| PPP4R2 | Hypothetical protein of ancient origin | AW961597 | -3.64 | 0.0166 |
| CYP1B1 | Cytochrome P450, family 1, subfamily B, polypeptide 1. | NM_000104 | -3.65 | 0.0042 |
| FBLN1 | Fibulin 1 | NM_006486 | -3.67 | 0.0005 |
| S100A9 | S100 calcium binding protein A9 | NM_002965 | -3.85 | 0.0032 |
| BIRC3 | Baculoviral IAP repeat-containing 3 | NM_001165 | -3.85 | 0.0022 |
| LOC728215 | Hypothetical locus LOC728215 | L10374 | -3.88 | 0.0328 |
| CYB5R2 | Cytochrome b5 reductase 2 | NM_016229 | -3.88 | 0.0328 |
| CCL2 | Chemokine (C-C motif) ligand 2 | NM_002982 | -3.90 | 0.0009 |
| BDKRB2 | Bradykinin receptor B2 | NM_000623 | -3.93 | 0.0068 |
| KIAA1166 | Zinc finger C4H2 domain-containing protein | NM_018684 | -3.94 | 0.0130 |
| NNMT | Nicotinamide N-methyltransferase | NM_006169 | -3.96 | 0.0028 |
| AKT3 | Protein kinase B, gamma | NM_005465 | -4.00 | 0.0280 |
| C1orf59 | Chromosome 1, open reading frame 59 | NM_144584 | -4.06 | 0.0062 |
| PTX3 | Pentraxin-related gene, rapidly induced by IL-1 beta | NM_002852 | -4.08 | 0.0141 |
| IGFBP2 | Insulin-like growth factor binding protein 2 | NM_000597 | -4.14 | 0.0007 |
| FZD7 | Frizzled homolog 7 | NM_003507 | -4.19 | 0.0000 |
| CCL20 | Chrmokine (CC motif) ligand 20 | NM_004591 | -4.23 | 0.0123 |
| MPP1 | Membrane protein, palmitoylated 1 | NM_002436 | -4.23 | 0.0026 |
| DNAJC15 | DnaJ (Hsp40) homolog, subfamily C, member 15 | NM_013238 | -4.26 | 0.0029 |
| C2orf89 | Chromosome 2, open reading frame 89 | FLJ46467 | -4.27 | 0.0020 |
| CTAG1A | Cancer - testis antigen 1A | NM_139250 | -4.30 | 0.0009 |
| KLF9 | Kruppel-like factor 9 | NM_001206 | -4.33 | 0.0158 |
| SERPINB3 | Serpin peptidase inhibitor, clade B, member 3 | NM_006919 | -4.35 | 0.0021 |
| VNN1 | Vanin 1 | NM_004666 | -4.67 | 0.0077 |
| KCNJ12 | Potassium inwardly-rectifying channel, subfamily J, member 12 | NM_021012 | -4.79 | 0.0064 |
| STAT6 | Signal transducer and activator of transcription 6, IL-4 induced | NM_003153 | -5.19 | 0.0014 |
| LOC388279 | Hypothetical locus LOC388279 | AF275804 | -5.44 | 0.0157 |
| HS6ST2 | Heparin sulphate 6-O-sulfotransferase 2 | NM_001077188 | -5.86 | 0.0000 |
| CXCL1 | Chemokine (C-X-C motif) ligand 1 | NM_001511 | -5.91 | 0.0203 |
| LCN2 | Lipocalin 2 (oncogene 24p3) | NM_005564 | -5.91 | 0.0028 |
