# Supplementary material for: siRNA Knockdown of Ribosomal Protein Gene RPL19 Abrogates the Aggressive Phenotype of Human Prostate Cancer
Source: PLoS One. 2011 Jul 22;6(7):e22672. doi: 10.1371/journal.pone.0022672 (PMC3142177; doi:10.1371/journal.pone.0022672)
Supplement: Table S3 — Gene ontology terms. Gene ontology (GO) biological process terms found to be significantly associated with genes significantly differentially expressed after knockdown of RPL19 using hypergeometric tests. (DOCX) [file pone.0022672.s004.docx]

**Supporting Information Table S3 - Gene ontology (GO) biological process terms found**

**to be significantly associated with genes significantly differentially expressed after**

**knockdown of RPL19 using hypergeometric tests**

| **GO ID** | ***p* value** | **GO Term** |
| --- | --- | --- |
| GO:0048731 | 8.29x10^-06^ | [**System development**](http://www.godatabase.org/cgi-bin/amigo/go.cgi?view=details&search_constraint=terms&depth=0&query=GO:0048731) |
| GO:0051674 | 2.56 x10^-05^ | [**Localization of cell**](http://www.godatabase.org/cgi-bin/amigo/go.cgi?view=details&search_constraint=terms&depth=0&query=GO:0051674) |
| GO:0007155 | 4.08 x10^-05^ | [**Cell adhesion**](http://www.godatabase.org/cgi-bin/amigo/go.cgi?view=details&search_constraint=terms&depth=0&query=GO:0007155) |
| GO:0009605 | 8.34 x10^-05^ | [**Response to external stimulus**](http://www.godatabase.org/cgi-bin/amigo/go.cgi?view=details&search_constraint=terms&depth=0&query=GO:0009605) |
| GO:0048870 | 9.52 x10^-05^ | [**Cell motility**](http://www.godatabase.org/cgi-bin/amigo/go.cgi?view=details&search_constraint=terms&depth=0&query=GO:0048870) |
| GO:0007416 | 1.70 x10^-04^ | [**Synaptogenesis**](http://www.godatabase.org/cgi-bin/amigo/go.cgi?view=details&search_constraint=terms&depth=0&query=GO:0007416) |
| GO:0043062 | 2.09 x10^-04^ | [**Extracellular structure organization**](http://www.godatabase.org/cgi-bin/amigo/go.cgi?view=details&search_constraint=terms&depth=0&query=GO:0043062) |
| GO:0032502 | 2.11x10^-04^ | [**Developmental process**](http://www.godatabase.org/cgi-bin/amigo/go.cgi?view=details&search_constraint=terms&depth=0&query=GO:0032502) |
| GO:0065007 | 2.23 x10^-04^ | [**Biological regulation**](http://www.godatabase.org/cgi-bin/amigo/go.cgi?view=details&search_constraint=terms&depth=0&query=GO:0065007) |
| GO:0048729 | 2.34 x10^-04^ | [**Tissue morphogenesis**](http://www.godatabase.org/cgi-bin/amigo/go.cgi?view=details&search_constraint=terms&depth=0&query=GO:0048729) |
| GO:0006954 | 2.64 x10^-04^ | [**Inflammatory response**](http://www.godatabase.org/cgi-bin/amigo/go.cgi?view=details&search_constraint=terms&depth=0&query=GO:0006954) |
| GO:0032501 | 3.04 x10^-04^ | [**Multicellular organismal process**](http://www.godatabase.org/cgi-bin/amigo/go.cgi?view=details&search_constraint=terms&depth=0&query=GO:0032501) |
| GO:0050900 | 4.11 x10^-04^ | [**Leukocyte migration**](http://www.godatabase.org/cgi-bin/amigo/go.cgi?view=details&search_constraint=terms&depth=0&query=GO:0050900) |
| GO:0050794 | 4.97 x10^-04^ | [**Regulation of cellular process**](http://www.godatabase.org/cgi-bin/amigo/go.cgi?view=details&search_constraint=terms&depth=0&query=GO:0050794) |
| GO:0007154 | 6.20 x10^-04^ | [**Cell communication**](http://www.godatabase.org/cgi-bin/amigo/go.cgi?view=details&search_constraint=terms&depth=0&query=GO:0007154) |
| GO:0016339 | 6.94 x10^-04^ | [**Calcium-dependent cell-cell adhesion**](http://www.godatabase.org/cgi-bin/amigo/go.cgi?view=details&search_constraint=terms&depth=0&query=GO:0016339) |
| GO:0006576 | 7.07 x10^-04^ | [**Biogenic amine metabolic process**](http://www.godatabase.org/cgi-bin/amigo/go.cgi?view=details&search_constraint=terms&depth=0&query=GO:0006576) |
| GO:0048522 | 7.40 x10^-04^ | [**Positive regulation of cellular process**](http://www.godatabase.org/cgi-bin/amigo/go.cgi?view=details&search_constraint=terms&depth=0&query=GO:0048522) |
| GO:0051050 | 9.13 x10^-04^ | [**Positive regulation of transport**](http://www.godatabase.org/cgi-bin/amigo/go.cgi?view=details&search_constraint=terms&depth=0&query=GO:0051050) |
| GO:0015939 | 9.38 x10^-04^ | [**Pantothenate metabolic process**](http://www.godatabase.org/cgi-bin/amigo/go.cgi?view=details&search_constraint=terms&depth=0&query=GO:0015939) |
