# Supplementary material for: siRNA Knockdown of Ribosomal Protein Gene RPL19 Abrogates the Aggressive Phenotype of Human Prostate Cancer
Source: PLoS One. 2011 Jul 22;6(7):e22672. doi: 10.1371/journal.pone.0022672 (PMC3142177; doi:10.1371/journal.pone.0022672)
Supplement: Table S4 — KEGG pathways. KEGG pathways containing genes significantly differentially expressed after RPL19 knockdown using hypergeometric tests. (DOCX) [file pone.0022672.s005.docx]

**Supporting Information Table S4 - KEGG pathways containing genes significantly**

**differentially expressed after *RPL19* knockdown using hypergeometric tests**

| **KEGG ID** | ***p* value** | **Term** |
| --- | --- | --- |
| 4060 | 0.002 | [Cytokine-cytokine receptor interaction](http://www.genome.jp/dbget-bin/www_bget?path:hsa04060) |
| 5222 | 0.004 | [Small cell lung cancer](http://www.genome.jp/dbget-bin/www_bget?path:hsa05222) |
| 920 | 0.005 | [Sulfur metabolism](http://www.genome.jp/dbget-bin/www_bget?path:hsa00920) |
| 330 | 0.005 | [Arginine and proline metabolism](http://www.genome.jp/dbget-bin/www_bget?path:hsa00330) |
| 5200 | 0.01 | [Pathways in cancer](http://www.genome.jp/dbget-bin/www_bget?path:hsa05200) |
| 250 | 0.015 | [Alanine, aspartate and glutamate metabolism](http://www.genome.jp/dbget-bin/www_bget?path:hsa00250) |
| 4510 | 0.016 | [Focal adhesion](http://www.genome.jp/dbget-bin/www_bget?path:hsa04510) |
| 4062 | 0.022 | [Chemokine signalling pathway](http://www.genome.jp/dbget-bin/www_bget?path:hsa04062) |
| 460 | 0.022 | [Cyanoamino acid metabolism](http://www.genome.jp/dbget-bin/www_bget?path:hsa00460) |
| 4810 | 0.027 | [Regulation of actin cytoskeleton](http://www.genome.jp/dbget-bin/www_bget?path:hsa04810) |
| 910 | 0.034 | [Nitrogen metabolism](http://www.genome.jp/dbget-bin/www_bget?path:hsa00910) |
| 5210 | 0.037 | [Colorectal cancer](http://www.genome.jp/dbget-bin/www_bget?path:hsa05210) |
| 5410 | 0.039 | [Hypertrophic cardiomyopathy (HCM)](http://www.genome.jp/dbget-bin/www_bget?path:hsa05410) |
