# Supplementary material for: siRNA Knockdown of Ribosomal Protein Gene RPL19 Abrogates the Aggressive Phenotype of Human Prostate Cancer
Source: PLoS One. 2011 Jul 22;6(7):e22672. doi: 10.1371/journal.pone.0022672 (PMC3142177; doi:10.1371/journal.pone.0022672)
Supplement: Table S5 — Pathways modulated after RPL19 knockdown. Top five interlinked pathways containing genes significantly differentially expressed after RPL19 knockdown using hypergeometric tests. (DOCX) [file pone.0022672.s006.docx]

**Supporting Information Table S5 - Top five interlinked pathways containing genes significantly differentially expressed after *RPL19* knockdown using hypergeometric tests**

| **ID** | **Molecules in Network** | **Score**  **(p = 10E^-Score^)** | **Focus Molecules** | **Top Functions** |
| --- | --- | --- | --- | --- |
| 1 | APOL3 (includes EG:80833), CASP1, CCDC50, CCL20, CHPT1, CUL5, DTX3, FRYL, HMCN1 (includes EG:83872), HOXB13, IGSF3, IL15, Interferon alpha, KYNU, LGALS3BP, NFKB2, NFKBIA, NFYB, peptidase, PP1/PP2A, PPP1CB, PPP2R3A, PPP2R5C, PSMB8, RNF144B, SLPI, SYNPO, TM7SF3, TPP1, TRIM31, UBE2D3 (includes EG:7323), UBE2L6, Ubiquitin, USP15, ZNF323 | 43 | 31 | Dermatological Diseases and Conditions, Hematological Disease, Immunological Disease |
| 2 | AKT3, BDKRB2, CD24, CD59, EPHA, EPHA5, EPHA7, ERBB3, G alpha, G protein beta gamma, G-protein beta, GAB1, GAS5, GNA14, GNAQ, GNAS, GNG7, Gpcr, IGF1R, IGFBP2, KCNJ6, LCN2, LPAR1, MET, NGEF, PA2G4, PDK1, PI3K, PIK3C2A, PLC, Pld, Shc, STAT, Trk Receptor, ULBP2 | 29 | 24 | Cellular Movement, Cell Morphology, Cellular Development |
| 3 | Actin, Alpha catenin, ARRB1, Cadherin, CALD1, Calmodulin, CASD1, CDH1, CDH7, CDK4, CLCN5, Cofilin, Cyclin A, Cyclin E, DPYSL2, EPB41L1, ERK, F Actin, FN1, IQGAP1, KCNQ2, LPP, MAP1B, OPHN1, PP2A, RAB8B, Rb, RPS2, SLC12A2, STXBP5, TMOD3, TPM1, TTN, Tubulin, ZYX | 27 | 23 | Cellular Assembly and Organization, Cellular Function and Maintenance, Skeletal and Muscular System Development and Function |
| 4 | ADCYAP1, BEX2, C3, CHEMOKINE, CXCL1, CXCL2, CXCL3, CXCL6, CXCL16, Ifn, IFN TYPE 1, IFNŒ±/Œ≤, Ikk (family), IL-1R, IL15RA, IRF, IRF5, ISL1, Itgal-Itgb2, LMO2, NFkB (complex), NfkB-RelA, NFKBIZ, PI3, Pro-inflammatory Cytokine, PTX3, RFTN1, RHOH, S100A9, SSBP2, TBK1, Tlr, TLR3, TNFAIP2, TNFSF13B | 27 | 23 | Cellular Movement, Hematopoiesis, Immune Cell Trafficking |
| 5 | 26s Proteasome, AKAP12, ATG5 (includes EG:9474), BIK, BIRC3, Caspase, CCNL2, CRIP2, DKK1, DNAJC14, DNAJC15, EPAS1, ERCC6, Estrogen Receptor, FUBP1, Histone h3, Histone h4, Hsp70, Hsp90, Hsp22/Hsp40/Hsp90, HSPB8, JAG2, LOXL3, NAP1L1, NR3C2, P38 MAPK, PTGES3 (includes EG:10728), RNA polymerase II, SERTAD2, SNAI1, TNIP1, TP63, UBE2, Vegf, ZNF83 | 27 | 23 | Cell Death, Hair and Skin Development and Function, Organ Development |
