# Supplementary material for: siRNA Knockdown of Ribosomal Protein Gene RPL19 Abrogates the Aggressive Phenotype of Human Prostate Cancer
Source: PLoS One. 2011 Jul 22;6(7):e22672. doi: 10.1371/journal.pone.0022672 (PMC3142177; doi:10.1371/journal.pone.0022672)
Supplement: Table S6 — Gene ontology molecular function terms. Gene ontology (GO) molecular function terms significantly associated with genes differentially expressed after knockdown of RPL19 using hypergeometric tests. (DOCX) [file pone.0022672.s007.docx]

**Supporting Information Table S6 - Gene ontology (GO) molecular function terms**

**significantly associated with genes differentially expressed after**

**knockdown of RPL19 using hypergeometric tests**

| **GO ID** | ***p* value** | **Term** |
| --- | --- | --- |
| GO:0008009 | 5.28x10^-05^ | [Chemokine activity](http://www.godatabase.org/cgi-bin/amigo/go.cgi?view=details&search_constraint=terms&depth=0&query=GO:0008009) |
| GO:0004062 | 1.07 x10^-04^ | [Aryl sulfotransferase activity](http://www.godatabase.org/cgi-bin/amigo/go.cgi?view=details&search_constraint=terms&depth=0&query=GO:0004062) |
| GO:0001664 | 1.15 x10^-04^ | [G-protein-coupled receptor binding](http://www.godatabase.org/cgi-bin/amigo/go.cgi?view=details&search_constraint=terms&depth=0&query=GO:0001664) |
