# Supplementary material for: siRNA Knockdown of Ribosomal Protein Gene RPL19 Abrogates the Aggressive Phenotype of Human Prostate Cancer
Source: PLoS One. 2011 Jul 22;6(7):e22672. doi: 10.1371/journal.pone.0022672 (PMC3142177; doi:10.1371/journal.pone.0022672)
Supplement: Table S7 — Glycosyltransferase and ion-channel genes modulated following RPL19 knockdown. Following RPL19 knockdown, modulated expression of only two glycosyltransferase genes was detected but with more profound changes to ion channels indicating significant changes to cellular homeostasis. (DOCX) [file pone.0022672.s008.docx]

**Supporting Information Table S7 - Glycosyltransferase and ion-channel**

**genes modulated following RPL19 knockdown**

| **Gene Name** | **Protein type** | **Fold change** | **Adjusted *p* value** |
| --- | --- | --- | --- |
| ***Glycosyltransferase genes:*** | | | |
| MGAT4A | Mannosyl (α-1,3)-glycoprotein β-1,4-N-acetylglucosaminyltransferase | -3.8 | < 0.05 |
| GALNACT-2 | Chondroitin sulfate N-acetylgalactosaminyltransferase-2 | 2.5 | < 0.05 |
| ***Potassium channel genes:*** | | | |
| KCNQ2 | Potassium channel, voltage-gated, KQT-like subfamily, alpha subunit, member 2 | -3.5 | < 0.05 |
| KCNJ6 | Potassium channel, inwardly-rectifying, alpha subunit, subfamily J, member 6 | 5.5 | < 0.01 |
| KCNJ12 | Potassium channel, inwardly-rectifying, alpha subunit, subfamily J, member 12 | -25 | < 0.01 |
| KCNAB2 | Potassium channel, voltage-gated, Shaker-related subfamily, beta subunit, member 2 | -2.25 | < 0.05 |
| ***Sodium channel genes:*** | | | |
| SCN3A | Sodium channel, voltage-gated, alpha subunit, subtype Nav1.3, | 9 | < 0.005 |
| SCN9A | Sodium channel, voltage-gated, alpha subunit, subtype Nav1.7, | 2.4 | <0.05 |
| ***Chloride channel genes:*** | | | |
| CLCN4 | Chloride channel / H^+^ antiporter, voltage-gated, subtype 4 | 2.1 | < 0.05 |
| CLCN5 | Chloride channel / H^+^ antiporter, voltage-gated, subtype 5 | 1.8 | < 0.05 |
