# Supplementary material for: siRNA Knockdown of Ribosomal Protein Gene RPL19 Abrogates the Aggressive Phenotype of Human Prostate Cancer
Source: PLoS One. 2011 Jul 22;6(7):e22672. doi: 10.1371/journal.pone.0022672 (PMC3142177; doi:10.1371/journal.pone.0022672)
Supplement: Table S8 — Characteristics of antibodies used to analyze changes in proteins expressed following RPL19 knockdown. Details of protein expression by Western Blotting analysed using a range of mono-specific antibodies to define changes in cellular phenotype following RPL19 knockdown. (DOCX) [file pone.0022672.s009.docx]

**Supporting Information Table S8 - Characteristics of antibodies used to analyze**

**changes in proteins expressed following *RPL19* knockdown**

| **Species** | **Protein/gene** | **Source** | **Catalogue #** | **Dilution/ Time** | **Results** |
| --- | --- | --- | --- | --- | --- |
| RP | AGR2 | Abcam PLC, Cambridge Science Park, Cambridge, UK | Ab43043 | 1:500 - O/N | Single band: 20kDa |
| RP | ERBB2 | Dako Denmark A/S, DK-2600, Glostrup, Denmark | A0485 | 1:1000 - 1 hour | Single band: 185kDa |
| RP | Cleaved CASP3 | Cell Signalling Technology, Danvers, MA 01923, USA | #9661 | 1:1000 - O/N | No band detected |
| RP | Cleaved CASP9 | Cell Signalling Technology, Danvers, MA 01923, USA | #9501 | 1:1000 - O/N | No band detected |
| RP | FABP5 | Hycult Biotech, Cambridge Bioscience, UK | HP9030 | 1:500 - 1 hour | Single band: 14kDa |
| RP | GIRK2/KCNJ6 | Sigma, St. Louis, MO 63101, USA | P8122 | 1:800 - O/N | Multiple bands including 47kDa |
| MM | MMP-1 | TCS Cellworks Ltd, Botolph, Claydon MK18 2LR, UK | ZIF-7101 | 1:1000 - 1 hour | Two bands: 120 & 54kDa |
| MM | MMP-13 | Neomarkers, Fremont, CA, USA | MS-826-P1 | 1:1000 - 1 hour | No bands detected |
| MM | MMP-3 | Neomarkers, Fremont, CA, USA | MS-810-P1 | 1:1000 - 1 hour | Many bands, none: 54kDa |
| MM | OPN | Santa Cruz, CA 95060, U.S.A. | SC-21742 | 1:200 - O/N | No bands detected |
| RP | PKCZ | Santa Cruz, CA 95060, U.S.A. | SC-216 | 1:200 - O/N | Single band: 75kDa |
| MM | RPL19 | Abnova (Taiwan) Corporation, Taipei, Taiwan | H00006143-M01 | 1:5000 - 1 hour | Single band: 25kDa |
| RP | S100A4 | Dako Denmark A/S, DK-2600, Glostrup, Denmark | A5114 | 1:700 - O/N | Two bands: 60kDa & 9kDa |
| MM | SCN3A | Sigma, St. Louis, MO 63101, USA | WH0006328-M1 | 1:500 - O/N | Multiple bands including 37kDa |
| MM | GAPDH | Sigma, St. Louis, MO 63101, USA | G8795 | 1:5000 - 1 hour | Single band: 37kDa |
| MM | Beta Actin | Sigma, St. Louis, MO 63101, USA | A5316 | 1:40000 - 30 mins | Single band: 42kDa |
